# Use of Three-Dimensional Molecular Descriptors to Predict the Glass Transition Temperature of Polymers

**DOI:** 10.3390/polym18111335

**Published:** 2026-05-28

**Authors:** Heitor Luiz Ornaghi Jr., Matheus de Prá Andrade, Lìdia Kunz Lazzari, Ademir José Zattera

**Affiliations:** Programa de Pós-Graduação em Engenharia de Processos e Tecnologias (PGEPROTEC), Universidade de Caxias do Sul, Rua Francisco Getúlio Vargas 1130, Petrópolis, Caxias do Sul 95070-560, RS, Brazil; mpandrade@ucs.br (M.d.P.A.); lklazzar@ucs.br (L.K.L.); ajzatter@ucs.br (A.J.Z.)

**Keywords:** molecular descriptors, modeling, structure–property relationship

## Abstract

In the present study, we built several models based on three-dimensional molecular descriptors to predict the glass transition temperature using a data set of 117 polymers. A data set division was established (training and test data) and consequently the models were developed and validated. Finally, the prediction/screen of the top models were compared. Three main descriptors were obtained with excellent predictions: E2 (E2u and E2s), which encodes angular and radial information about atomic configuration, usually in relation to two atoms; TDB (TDB10u, TDB10e, TDB10s) describes the relationship between the average three-dimensional (Euclidean) distance and the topological distance (path length, or number of bonds) between possible atom pairs in a molecule; and RDF (RDF25i, RDF65u, RDF25u) describes the density of atoms at different distances from a reference atom, capturing information about the local structure of the molecule. An initial exploratory GA-LDA classification analysis using 3D descriptors revealed only partial discrimination between polymers with distinct T_g_ behavior, indicating that simplified 3D structural representations alone are limited for robust T_g_ prediction. Consequently, graph-based (2D) descriptors models were created and the prediction of the T_g_ was successfully achieved. Overall, the most influential variables are predominantly graph-based (2D) descriptors associated with molecular connectivity patterns (e.g., autocorrelation-type descriptors such as ATS2*), topological/shape-related indices (TSC* family), and ring-related terms. This attribution profile is consistent with the expected physicochemical determinants of the glass transition: polymer repeat units with higher structural rigidity, constrained connectivity, and increased ring/unsaturation content that typically exhibits reduced segmental mobility and, therefore, higher T_g_.

## 1. Introduction

The glass transition temperature (T_g_) is an important characteristic of the polymer that governs the maximum service temperature of the polymer [1,2]. T_g_ can be defined as a temperature range in which the material is converted from a brittle solid to a softer material followed by a higher molecular mobility [3]. It is mainly governed by the amorphous segments of the polymeric chains (that is the reason why a hypothetically 100% crystalline polymer does not present this transition) [4]. The crystalline portions (also known as crystallites) do not affect (at least considerably) the properties below T_g_. At temperatures below T_g_ the properties are mainly governed by the intermolecular forces and by the way the chains are packed [5,6]. As temperature is given to the system, the increase in the internal energy allows different chain conformations at the same energy level, allowing a deformation of the amorphous component and drastically altering some properties such as specific heat (increasing), modulus (decreasing) and expansion (increasing). If, hypothetically, the polymer does not have amorphous regions, no T_g_ will be presented. Considering that there are abrupt changes in some properties at T_g_ and that this transition is a transition of the amorphous part (T_m_ is a transition of the crystalline portion of the polymer), the prediction of it is vital during the selection of materials during different applications.

There are some discussions about T_g_ be a thermodynamic or kinetic transition [7], but the fact is that when experimentally measured by different techniques, different results can be found for the same polymer [8]. In spite of some standardizations found in the literature, the main problem is that many parameters such as structural, conformational, or constitutional features besides molecular weight and experimental conditions (heating rate, pressure, clamp (in the case of dynamic mechanical thermal analysis)), etc.) highly influence the T_g_ value. Also, T_g_ measures demands time, appropriate equipment and cost. To overcome these problems, different models are employed in the literature. Among these models, quantitative structure–property relationship (QSPR) modeling has been extensively used for the prediction of polymer properties [9] that use molecular descriptors (mathematical representation of molecules’ properties that are generated by algorithms) and methods of derivation (including multiple linear and nonlinear relationships) to determine different properties. In this model, different properties can be evaluated as follows: (i) properties of single molecules (boiling point, critical temperature, and vapor pressure. for example), (ii) interactions between different molecular species (octanol-water partition coefficient, the aqueous solubility of gases and vapor, solvent polarity scales, for instance), (iii) surfactant properties (critical micelle concentration, cloud point. for example), and (iv) properties of polymers (glass transition temperature (T_g_), refractive index, rubber vulcanization acceleration, for instance). Several contributions regarding each topic were explored in the literature with distinct methodologies. For example. Katritzky et al. [9] studied a QSPR based on a Comprehensive Descriptors for Structural and Statistical Analysis (CODESSA) to rationalize the T_g_ of 22 specific polymers. According to the authors, the results can be further used for the prediction of T_g_ values for unknown polymers of similar structures. However, QSPR models may be restricted to a dataset in which aspects such as overall accuracy, Applicability Domain, and the chance of correlation have to be satisfactory to a regulatory acceptance [10].

Some studies can be found on the literature regarding the prediction of T_g_ for different polymers. Brostow et al. [11] presented an equation for the prediction of T_g_ in binary blends and copolymers considering the miscibility of the components. Pilania et al. [12] uses machine learning-based predictive modeling of glass transition temperatures for polyhydroxyalkanoate homopolymers and copolymers. The authors validated a statistical learning model to allow for a mapping of the polymer fingerprints onto the property space in a physically meaningful and reliable manner. In spite of a rapid prediction of the T_g_, some uncertainties are still presented in the model. Khan et al. [13] used QSPR modeling for the prediction of the T_g_ of diverse polymers using 2D molecular descriptors from a single repeating unit. Six descriptors were obtained and validated by different methods. The authors claim that the main factors that influence an increase in T_g_ are dipole moment, polarizability, aromaticity, branching in polymer chain, and heteroatom count. Petrosyan et al. [14] models T_g_ for polymeric coating materials, applying a QSPR mixture-based approach. The authors presented new techniques to structurally represent block copolymers using DRAGON descriptors. The model ranged from 0.851 to 0.911 for the training set and the most influencing factor was the octanol-water partition coefficient and 3D-MoRSE unweighted descriptors. Zhang and Xu [15] used machine learning for the prediction of T_g_ using the molecular traceless quadrupole moment and molecule average hexadecapole moment as descriptors to predict T_g_. Sixty different polymers were used to model T_g_. Chen et al. [16] studied the computational structure–property relationship of T_g_ for a diverse set of polymers using 80 polymers to build a structure–property relationship (QSAR). The authors create several predictive models using from 1 to 10 physicochemical variables. The best model was the one using seven descriptors indicating that several factors are responsible for T_g_. The correlation coefficient of r2 = 0.77 was found in this study. Higuchi et al. [17] studied T_g_ prediction of linear homo/heteropolymer and cross-linked epoxy resins. The highest accuracy (Q2 = 0.920, RMSE = 34.3 over the training set of 270 polymers; R2 = 0.779, RMSE 35.9 K for an external test set of 119 polymers) was found by the authors in the 12 x repeated three-fold cross-validation challenge. Karuth et al. [18] applied cheminformatics and molecular dynamics simulations (CG-MD—coarse-grained molecular dynamics) for the prediction of the T_g_ of amorphous polymers. The authors grouped the molecular features from the machine learning model into cohesive energy, chain stiffness, and grafting density. Hundreds of polymers were used as the data set and it was identified that the higher intermolecular interaction and chain stiffness increase the T_g_ of CG polymers, where their relative influences are coupled with the existence of side chains grafted on the backbone.

The aim of this study is to develop new QSPR models to predict the glass transition temperature for a series of 117 polymers using 3D Chemopy descriptors. Finally, the consensus model selected was used for the prediction of a true external set of compounds to confirm the predictive ability of the developed model. Finally, a regression model using the developed models was used to predict the glass transition behavior of the polymer series. Also, a structure vs. property relationship is discussed considering the test set of polymers.

## 2. Materials and Methods

### 2.1. Polymer Data Set

**Figure 1:**
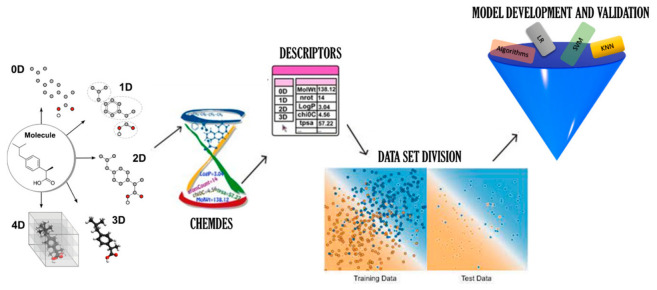
Schematic representation of the model development.

A data set of 117 polymers with the glass transition temperature values was obtained from [19,20] (found on the Supplementary Material File S1, Table S1). The data comprises a series of organic polymers with the respective chemical structure, SMILES (Simplified Molecular Input Line Entry System) notation and T_g_ values. Same methodology from [13] was used in this study (schematically represented on Figure 1).

### 2.2. Descriptor Computation and Data Set Division

3D Chemopy molecular descriptors (the database containing more 3D descriptors in the library) were used, totalizing 502 molecular descriptors. The molecular descriptors used were: geometric descriptors (12), CPSA descriptors (30), WHIM descriptors (70), RDF descriptors (180), MoRSE descriptors (210). All molecular descriptor details can be found from the library available in [21]. Also, the calculations were performed using DTC-QSAR software freely available in [21].

The Classification QSAR approach (with Positive and Negative group notations) was used, with a data pre-treatment (variance cut-off—0.001; inter R2 cut-off—0.99). The data pre-treatment was used to remove constant and inter-correlated descriptors. The less-discriminating descriptors (for removal descriptors that enhance noise in the data with less or no contribution) were also used for comparison. After data pre-treatment, the dataset was divided into training and test sets, where 70% of compounds were selected for training. The training set is employed for model development and model selection (next steps), while the test set will be exclusively employed for evaluation of selected model (recommended for dataset with 50–500 chemicals). Consequently, the training and test set were divided using a rational approach using the Kennard–Stone’s algorithm. GA-LDA (Genetic Algorithm–Linear Discriminant Analysis) was employed as the chosen model development method.

Two-dimensional (2D) molecular descriptors were calculated from the polymer repeating-unit structures using RDKit in combination with the Mordred descriptor framework, with the descriptor calculation restricted to 2D (graph-based) information. This approach yields a comprehensive descriptor space that represents the molecular constitution and connectivity of the repeat unit, which is particularly relevant for the polymer QSPR because T_g_ is strongly influenced by factors such as chain rigidity, functional group chemistry, polarity, and overall topological complexity.

The generated descriptors cover several families commonly employed in quantitative structure–property relationship studies, including constitutional descriptors (composition and size-related indices), topological/connectivity descriptors (graph distance and adjacency-based indices), electrotopological-state (E-state) descriptors, information-content and complexity measures, autocorrelation descriptors, and fragment/count-based descriptors.

### 2.3. Prediction

Before continuous T_g_ regression modeling, an exploratory classification workflow was employed to investigate whether broad T_g_ tendencies (low-T_g_ vs high-T_g_ behavior) could be discriminated using a reduced set of 3D descriptors. For this purpose, T_g_ values were discretized into Positive (P) and Negative (N) classes according to the median T_g_ value of the dataset, ensuring approximately balanced classes and minimizing class imbalance effects during LDA training. This preliminary step was intended exclusively to evaluate the discriminatory capacity of 3D descriptors under simplified response-space conditions and not as the final predictive framework for T_g_ estimation. The Classification QSAR approach method was selected for all the descriptors [13]. The Training set file (from FIRST PART) and a Query file were used to predict the model using the Two-class LDA model development technique. Also, the polymers were divided into Positive (P) and Negative (N) groups.

The dataset was divided into 70% training and 30% test subsets using the Kennard–Stone rational selection procedure implemented in the DTC-QSAR platform. This partitioning strategy was used exclusively for the exploratory GA-LDA classification analysis and was not reused during the regression workflow described in Section 2.4.

The classification model was developed using the Two-class Linear Discriminant Analysis (LDA) approach available in the DTC-QSAR framework. The objective of this stage was not to establish the final predictive model for T_g_ estimation, but rather to evaluate the discriminatory capacity of simplified 3D descriptor representations under a reduced response-space scenario.

### 2.4. Regression Workflow for T_g_ Prediction

The regression workflow was developed independently from the exploratory GA-LDA classification analysis. Consequently, a completely separate data partitioning strategy was adopted exclusively for continuous T_g_ prediction using graph-based 2D descriptors.

Because the initial 2D descriptor space is high-dimensional, a structured workflow was adopted to obtain a robust regression model for predicting the glass transition temperature (T_g_). First, a two-stage feature selection protocol was applied to reduce redundancy and improve generalization. In Stage 1 (correlation screening), descriptors were ranked according to their absolute correlation with T_g_, and the 120 most-correlated descriptors were retained as candidates. In Stage 2 (PLS–VIP selection), a Partial Least Squares (PLS) regression model was fitted using these candidates and Variable Importance in Projection (VIP) scores were computed; only descriptors with VIP ≥ 1.0 were kept for machine learning, prioritizing variables with meaningful contribution to T_g_ while mitigating multicollinearity.

All selected descriptors were subsequently standardized using z-score scaling (zero mean and unit variance). Scaling parameters were determined from the training data and consistently applied to cross-validation folds and the external test set, ensuring numerical comparability among descriptors and preventing scale dominance during optimization, especially for kernel and ensemble models.

Four regression algorithms were then evaluated to cover distinct hypothesis classes widely used in engineering-oriented QSPR modeling: PLS regression (baseline latent-variable linear model), Support Vector Regression (SVR) (kernel-based nonlinear regression), Random Forest (RF) (bagging-based ensemble of decision trees), and Extreme Gradient Boosting (XGBoost) (boosting-based tree ensemble designed for high predictive performance on structured datasets). All models were implemented in Python, and hyperparameters were optimized on the training set using five-fold cross-validation (randomized search over predefined ranges), targeting minimization of RMSE and maximization of Q^2^. The final model choice was based on cross-validated and external test performance, while full model-to-model comparisons are reported in the Section 3.

Model validation followed a combined strategy. Initially, the dataset was split into 80% training and 20% external test partitions; the external test set was held out and used only for final evaluation. Within the training partition, a five-fold cross-validation procedure was performed to estimate internal predictivity and stability, yielding Q^2^ and RMSECV. After cross-validation, the selected model was refit using the full training set and then evaluated on the held-out test set. Performance was quantified using Q^2^, RMSECV, R^2^ (test), RMSE (test), MAE, and percentage MAE. External validation was additionally assessed using the Golbraikh–Tropsha criteria computed on the external test set (k, k′, r^2^_0_, r^2^_0_′, and Δr^2^).

Finally, model adequacy and interpretability were supported through diagnostic and explanatory analyses, including observed vs. predicted plots, calibration curves, residual diagnostics (residual difference plots) and Q–Q plots, as well as SHAP (SHapley Additive exPlanations) to quantify descriptor contributions and to relate dominant predictors to physicochemical and structural drivers of T_g_.

## 3. Results

Part I—Model Development

This module can be divided into three distinct categories: Section 3.1—Data Pre-Treatment; Section 3.2—Model Development and Validation; Section 3.3—Robustness Testing and Applicability Domain.

### 3.1. Data Pre-Treatment

The prepared input descriptor matrix with the respective data (before and after data set division) can be found on the Supplementary Material (Supplementary Material File S2). At this stage, the descriptors that were found constant and inter-correlated in the training set are removed from both the training and test sets.

### 3.2. Model Development and Validation

The best model results, the comparison among the top models, and the selected descriptors set for the selected model are presented here. All files generated by the calculations are presented in the Supplementary Material (Supplementary Material File S3).

(i) Best model results comprises the basic information for the selected model (GA-LDA) as a fitness score value, qualitative validation metrics (for both training and test set), and tables (Tables S2 and S3, found in Supplementary Material File S4) comprising some information about the training and test set.

**Figure 2:**
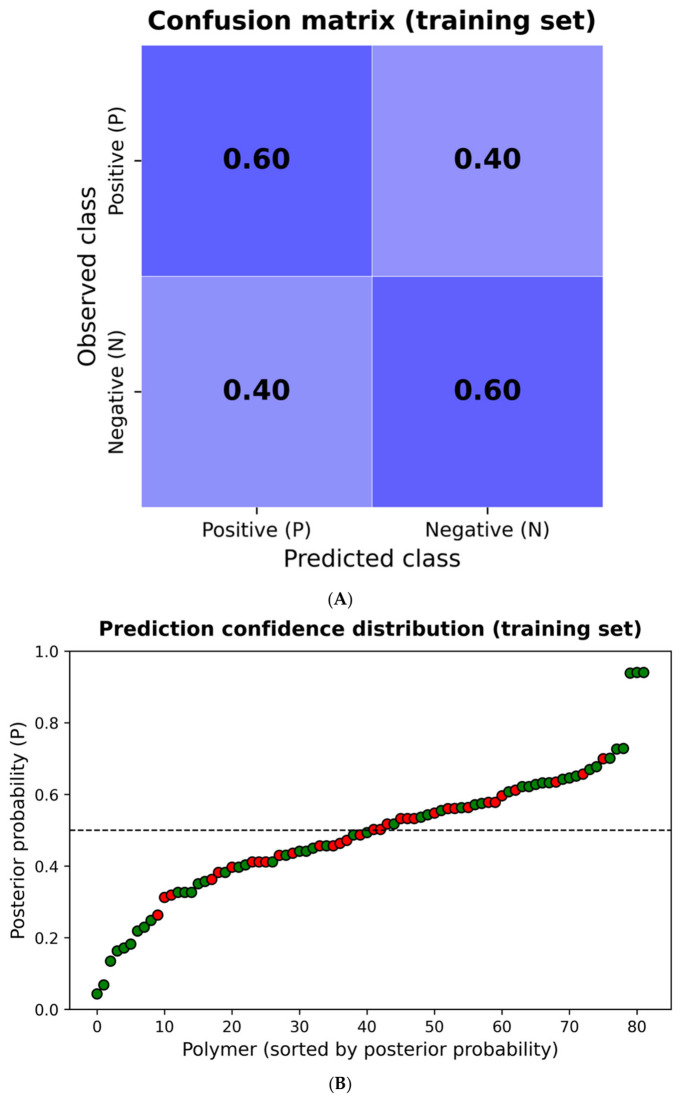
(**A**) Normalized confusion matrix of the GA-LDA model for the training set. (**B**) Prediction confidence distribution of the GA-LDA model for the training set. Each point represents one polymer, ordered by its posterior probability for the Positive class (P). Green points correspond to correctly classified polymers, while red points indicate misclassifications. The dashed line at 0.5 represents the decision threshold separating the P and N classes.

**Figure 3:**
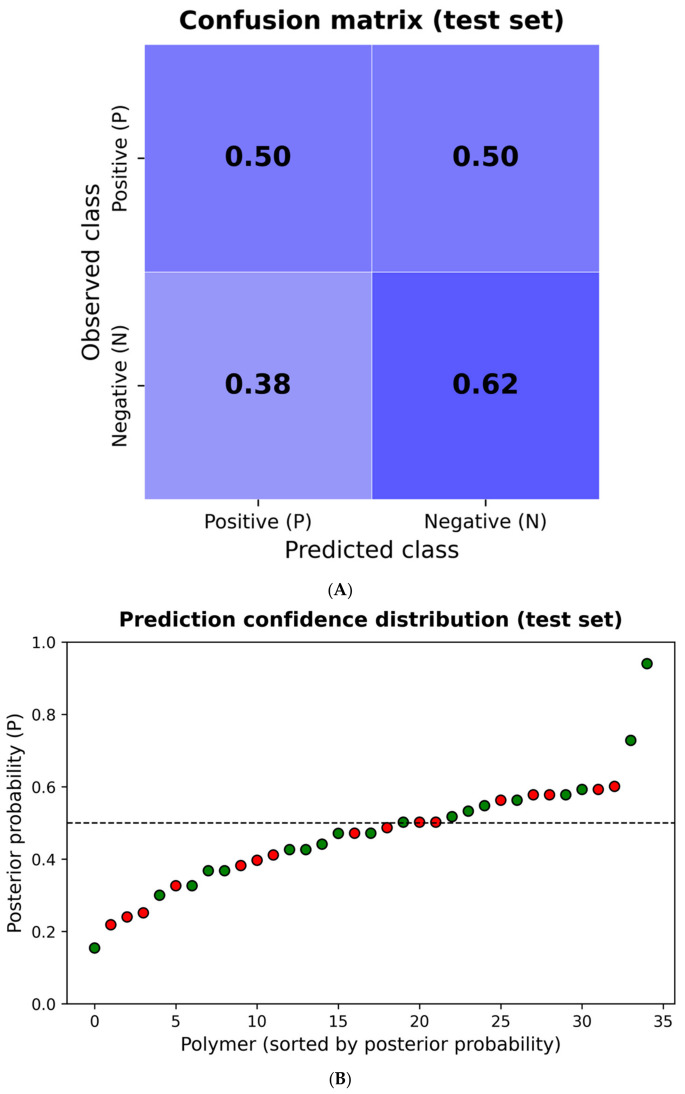
(**A**) Normalized confusion matrix of the GA-LDA model for the test set. (**B**) Prediction confidence distribution of the GA-LDA model for the test set. Each point represents one polymer, ordered by its posterior probability for the Positive class (P). Green points correspond to correctly classified polymers, while red points indicate misclassifications. The dashed line at 0.5 represents the decision threshold separating the P and N classes.

The final GA-LDA model resulted in a fitness score (using the training set only) of −0.6946, with a Wilks’ Lambda (train) of 0.8473 and a Wilks’ Lambda (test) of 0.8239. The validation metrics for the training and test sets are presented in Table 1 while the training and test set are presented on Figure 2A,B and Figure 3A,B, respectively. The data from Figure 2 are presented in the Supplementary Material File S4 (Tables S2–S4).

Both Positive and Negative polymer classes were correctly classified in 60% of cases, indicating balanced performance.

For both series (training and test), a balanced performance is observed among all polymers tested and similar results were obtained. The confusion matrix indicates a value of 0.60 and 0.50 for Positive/Positive (True Positive), considering training and test series, respectively. The other Real Positive condition (False Negative) was 0.40 and 0.50, indicating this amount of miss/underestimation. For the Real Negative conditions (False Positive and True Negative), the values obtained were 0.40 and 0.60 for the training series and 0.38 and 0.62 for the test series, respectively. For the former series, a false alarm/overestimation is considered while for the latter a correct rejection is considered.

For Figure 2 and Figure 3B, each point represents a polymer, ordered according to the predicted probability on the *x*-axis, while the *y*-axis indicates the posterior probability P(Pdescriptors). The green points correspond to correctly classified samples, and the red points indicate incorrect classifications. The dashed line at 0.5 represents the decision threshold that separates classes P and N. It can be observed that classification errors are concentrated near this threshold, while samples with more extreme probabilities exhibit greater confidence in the prediction, highlighting the uncertainty behavior of the model in boundary regions between classes [22].

(ii) Compare top models presents all the top GA-LDA models selected in the final generation/iteration. The redundant models are removed so the final number of top models may vary with each run. Table 2 presents only the top five models. All models are presented in the Supplementary Material File S3.

The descriptors’ category significances are described below [21,23]:

1. E2 (E2u and E2s) refers to a specific category of descriptors which encode angular and radial information about atomic configuration, usually in relation to two atoms.
2. TDB (TDB10u, TDB10e, TDB10s) describes the relationship between the average three-dimensional (Euclidean) distance and the topological distance (path length, or number of bonds) between possible atom pairs in a molecule.
3. RDF (RDF25i, RDF65u, RDF25u) describes the density of atoms at different distances from a reference atom, capturing information about the local structure of the molecule.

For further development, only Model Number 1 (the best model obtained) was considered. For Model Number 1, the training and test models are presented in Tables S3 and S4. For both tables, the response class (P or N) and the numerical values of the descriptors from Model Number 1 (Table 2) are presented. Tables S3 and S4 can be found in the Supplementary Material File S4.

The descriptors’ significances are described below [21,23]:

1. E2u: second component of accessibility directional WHIM index/unweighted.
2. TDB10u: a ring descriptor related to deviation/distance indices (D/Dtr) of order 10 (referred to as D/Dtr10). The suffix “u” likely indicates that it is the “unscaled” or “unnormalized” version of the description. D topological distance-based descriptors—lag 10 unweighted.
3. RDF25i: three-dimensional (3D) radial distribution function (RDF) descriptor that encodes information about the spatial distribution of atoms in a molecule, weighted by a specific type of atomic property. The suffix “i” indicates that the descriptor calculation is weighted by the first ionization potential of the atoms involved. The number “25” refers to a specific data point or “lag” (distance interval) within the series of calculated RDF descriptors (typically in increments of 0.5 Å, but the exact value refers to a position in the series, not necessarily 25 Å). Radial distribution function—025/unweighted.

**Figure 4:**
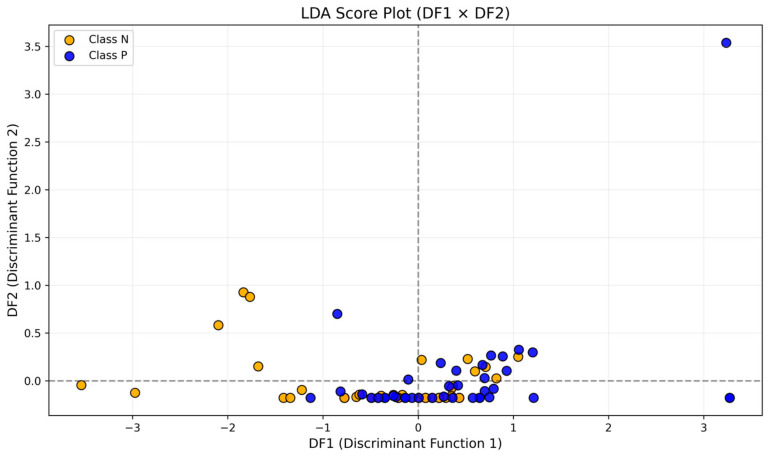
Discriminant functions from LDA score plot for N and P classes.

Figure 4 shows the LDA score plot based on DF1 (discriminant function 1) and an auxiliary DF2, which was derived from PCA (Principal Component Analysis), used for visualization purpose only, since binary LDA generates only one true discriminant function by nature. There is a clear trending of separation between N and P classes along DF1 axis. The class P polymers (blue) cluster is predominant in the positive DF1 region, whereas class N polymers (orange) concentrate mostly in negative DF1 values. In the center there is an overlap over the decision boundary.

The auxiliary DF2 axis reflects internal structural variability not directly related to class differentiation. A small number of samples appear as vertical outliers, indicating polymers with distinct 3D descriptor profiles. Overall, the plot confirms that DF1 captures the essential structural information responsible for class discrimination in the GA-LDA model.

To understand which structural features drive class separation in the GA–LDA model, the coefficients (loadings) of the discriminant function DF1 were analyzed. Since the system contains only two response classes (P and N), the LDA generates a single discriminant function, meaning that DF1 concentrates all discriminative information.

The loadings reveal a strong dominance of the descriptor E2u, which presents a coefficient of –7.10. This indicates that the discrimination between the two classes is governed primarily by energetic/electronic 3D characteristics captured by this descriptor. In contrast, the remaining descriptors selected by the genetic algorithm (TDB10u and RDF25i) show negligible contributions (–3.0 × 10^−5^ and 5.0 × 10^−6^, respectively), suggesting that these variables provide little incremental structural information to the classification.

Such behavior is common in binary LDA systems, where only one discriminant direction exists, and a single descriptor often dominates the mode [24]. The results indicate that DF1 is essentially a function of E2u, which aligns with the trends observed in the score plot and with the structural patterns present in Tables S5 and S6.

**Figure 5:**
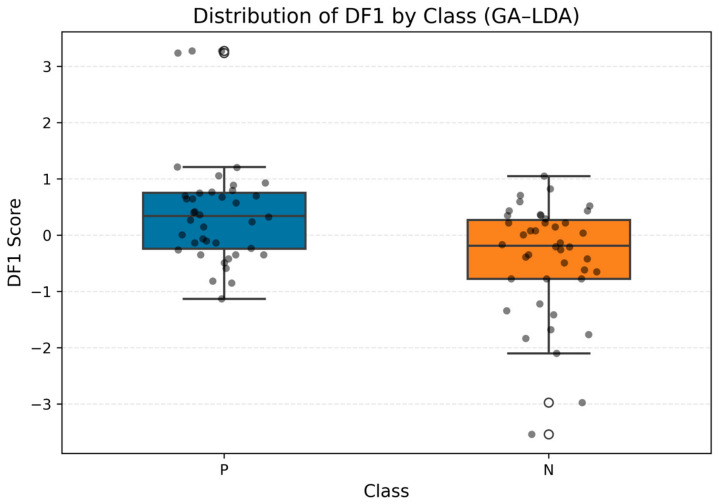
LDA score plot based on DF1, the single discriminant function obtained for this binary system.

To further characterize the internal behavior of GA-LDA model, the distribution of DF1 scores for classes P and N was analyzed (Figure 5). As already seen in Figure 4, class P polymers tend to appear on positive DF1 score and class N polymers on negative ones. Class P exhibited compact distribution that indicates structural homogeneity among the polymers associated with this class. Class N presented a wider dispersion and lower DF1 median, possibly reflecting greater structural variability.

Although the GA-LDA revealed partial structural separation between low- and high-T_g_ polymers, the moderate discrimination performance suggested that simplified binary classification based exclusively on 3D descriptors was insufficient to fully represent the complexity of T_g_ behavior across chemically heterogeneous polymer systems. Consequently, continuous regression-based QSPR models using graph-based 2D descriptors were subsequently developed to preserve the full quantitative information associated with T_g_.

### 3.3. Robustness Testing and Applicability Domain

The standard approach training and test as well as the Y-randomization are presented in Tables S7 and S8 and Table 3, respectively.

**Figure 6:**
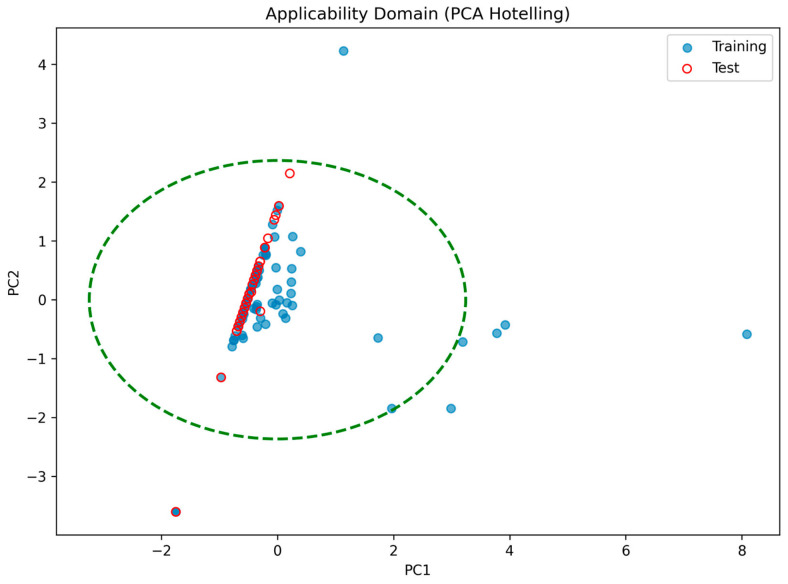
Applicability Domain (AD) determined using PCA and Hotelling’s T^2^ ellipse for the GA–LDA model. Blue points represent training set polymers and red points represent test set polymers. Samples located inside the ellipse fall within the model’s chemical space (inliers), while samples outside the boundary are considered out-of-domain and may yield less reliable predictions.

Figure 6 presents the Applicability Domain (AD) of the GA–LDA model, evaluated using Hotelling’s T^2^ method based on the first two principal components (PC1 and PC2) calculated from the training set. The green dashed ellipse represents the 95% confidence region that defines the chemical space learned by the model during training. Training polymers are shown as blue points, whereas test polymers are represented by red hollow markers.

Most of the test samples fall inside the confidence ellipse, indicating that they lie within the chemical space defined by the training descriptors and therefore belong to the valid AD of the model. A few samples appear close to the boundary or slightly outside the ellipse, suggesting limited structural extrapolation. These cases warrant more cautious interpretation but still provide valuable insight into the robustness of the GA–LDA model.

Tables S7 and S8 present the three descriptors (Model 1 from Table 2) with the respective values as well as the response and predicted class (P or N) and the status (Applicability Domain) (inside or outlier). A sample is considered inside the Applicability Domain if its characteristics (features or descriptors) are similar to the data used to train the model, ensuring the prediction is a form of interpolation. Conversely, a sample is an outlier (or out-of-domain) if it falls outside these boundaries, meaning its prediction would be an extrapolation and thus less reliable.

Inside the AD (Inlier): Data points within the AD are similar to the training data in the feature space. The model is expected to provide accurate and reliable predictions for these points because it has “seen” similar examples during training.

Outside the AD (Outlier): Data points outside the AD are considered outliers in the prediction context. Predictions for these samples are generally less reliable and should be treated with caution, as the model may not have learned the underlying relationships for these novel conditions.

In general, most polymers are inside the AD, indicating the reliability of the model used. Tables S5 and S6 can be found in Supplementary Material File S5.

**Figure 7:**
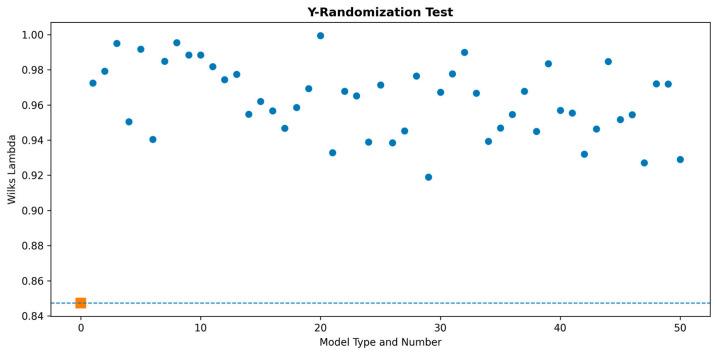
Y-randomization test results for the model. Scatter plot showing the Wilks’ Lambda values for the original model and for the 50 randomized models used in the Y-randomization test. The dashed line indicates our Wilks’ Lambda value being the orange square our result.

The Y-randomization test is presented in Figure 7. The original model value is 0.8473, the average Wilks’ Lambda from 50 random models is 0.9628, and the difference from random to original is −0.1155. This test is a validation technique to assess if a model is statistically significant or just a result of random chance. It works by comparing the performance of a model built on the original data (y) to models built on many shuffled versions of the (y) variable, while keeping the descriptor data (x) intact. If the original model performs significantly better than the randomized models, it indicates the model is not based on chance correlation.

Our results presented Wilks’ Lambda values for the original model, that are lower compared to the other 50 models, indicating that the present models are not developed by chance, as demonstrated by Ambure et al. [25]. All data can be found in Supplementary Material File S4 (Tables S3–S5).

Part II—Predict/Screen

In this part the predict class for query compounds was obtained using the input training model, in addition to the Applicability Domain for every query compound using the standardization approach and the posterior probabilities, which can help to judge the reliability for each prediction using the Concept of Confidence Estimation approach. Tables S7 and S8 (can be found in Supplementary Material File S5) showed the query results for the LDA results.

**Figure 8:**
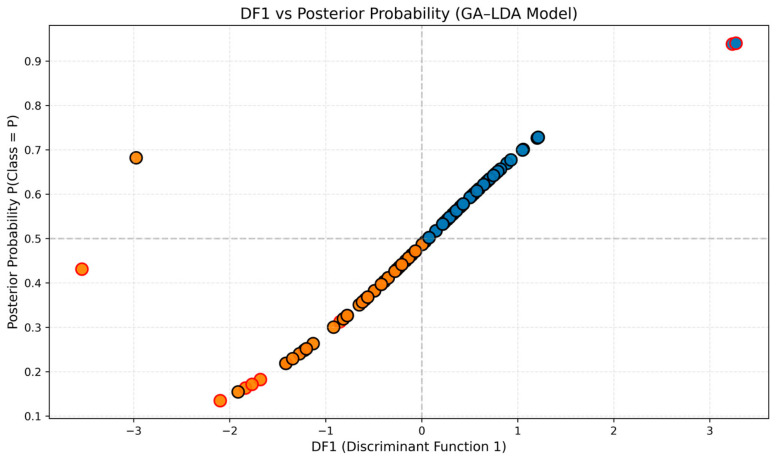
Relationship between DF1 and the posterior probability for class P obtained using the GA–LDA model for polymers without a known experimental response class (Table S7). Samples with DF1 > 0 exhibit posterior probabilities above 0.5 and are classified as P (blue), while samples with DF1 < 0 show probabilities below 0.5 and are classified as N (orange), consistent with the expected behavior of a linear discriminant function.

Figure 8 shows the relationship between DF1 and the posterior probability for class P for the polymers without the experimental response class (Table S7). As expected for a linear discriminant model, samples with positive DF1 values exhibit higher posterior probabilities and are assigned to class P, while samples with negative DF1 values show probabilities below 0.5 and are classified as N.

The clear monotonic trend between DF1 and posterior probability confirms the internal coherence of the GA–LDA model. Samples located outside the Applicability Domain (red-edged markers) appear closer to the decision boundary and display lower confidence, indicating that their predictions should be interpreted cautiously.

*Significance of the descriptors used in the QSAR models:*

1. E2u: Second component accessibility directional WHIM (Weighted Holistic Invariant Molecular) index/unweighted. WHIM descriptors are molecular descriptors based on statistical indices calculated on the projections of the atoms along principal axes. These descriptors are built in such a way as to capture relevant molecular 3D information regarding molecular size, shape, symmetry and atomic distribution with respect to invariant reference frames.
2. TDB10u: 3D topological distance-based autocorrelation—lag 10/unweighted.
3. RDF25i: Radial distribution function/weighted by relative first ionization potential.

It was certain that the model using similar polymers would lead to better predictive power, as mentioned in the studies proposed by refs. [11,12,13,14] (presented in the Introduction section). But our objective was to use the most different structures as possible to evaluate this new possibility. Our results did not show as positive when using 3D descriptors but a good fit was obtained using 2D descriptors, in spite of this variability of polymers. A future study is intended to measure the predictive power of polymers containing similar chemical structure.

Part III—Glass transition prediction

### 3.4. Regression Models Training and Evaluation

Four regression algorithms (PLS, SVR, Random Forest, and XGBoost) were evaluated using the same descriptor set and validation strategy. Table 3 summarizes the predictive performance obtained from five-fold cross-validation on the training set (Q^2^ and RMSECV) and from the external 20% test set (R^2^, RMSE, MAE, and percentage MAE). Importantly, it should be emphasized that this study follows a general, polymer class-agnostic strategy. In other words, the proposed model was developed to predict T_g_ across a chemically diverse set of polymers rather than be tailored to a single family (e.g., polyolefins, polyesters, or polyamides). While restricting the scope to a specific polymer class would typically yield higher apparent accuracy due to reduced structural variability, the broader formulation adopted here is more consistent with engineering screening scenarios in which the candidate materials span multiple chemistries.

SVR exhibited poor predictive ability (R^2^_test = 0.101), indicating that the selected descriptor space and/or kernel configuration did not provide a stable mapping for T_g_ in this dataset. Random Forest achieved intermediate performance (R^2^_test = 0.693), while PLS provided competitive cross-validated predictivity (Q^2^_cv = 0.621) but a weaker external test performance compared to XGBoost. The best overall results were obtained with XGBoost, which achieved the highest test-set accuracy (R^2^_test = 0.825) and the lowest prediction errors (RMSE_test = 31.515 K; MAE_test = 23.400 K; MAE% = 7.141%), while maintaining a strong cross-validated predictivity (Q^2^_cv = 0.612). Therefore, XGBoost was selected as the final regression model for subsequent diagnostic evaluation and interpretability analysis.

In addition to conventional predictive metrics, the external reliability of the selected regression model (XGBoost) was examined using the Golbraikh–Tropsha criteria, which are widely adopted in QSPR/QSAR studies to verify that the observed–predicted relationship is close to the ideal identity line and that systematic bias is limited. For the external test set, the slopes of the regressions through the origin were close to unity (k = 1.054 and k′ = 0.942), and the deviations associated with origin-constrained fits were small (r^2^_0_ = 0.869; r^2^_0_′ = 0.846; Δr^2^ = 0.024). These results support that the model predictions are not dominated by scaling artifacts and that the predictive relationship remains stable under stringent external-validation checks, reinforcing the suitability of the proposed approach for engineering-oriented polymer screening across chemically diverse structures.

### 3.5. Predictive Performance and Model Interpretability

**Figure 9:**
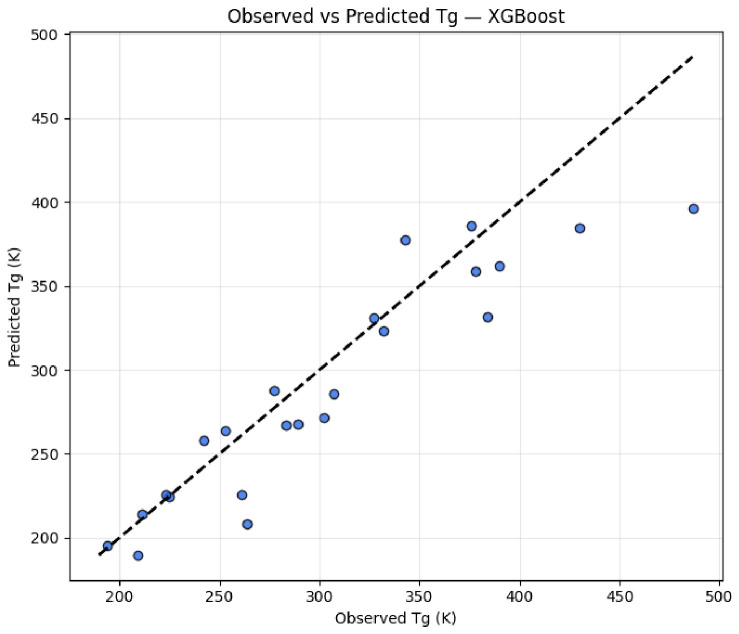
Predicted x observed T_g_ (K) for the polymers studied.

**Figure 10:**
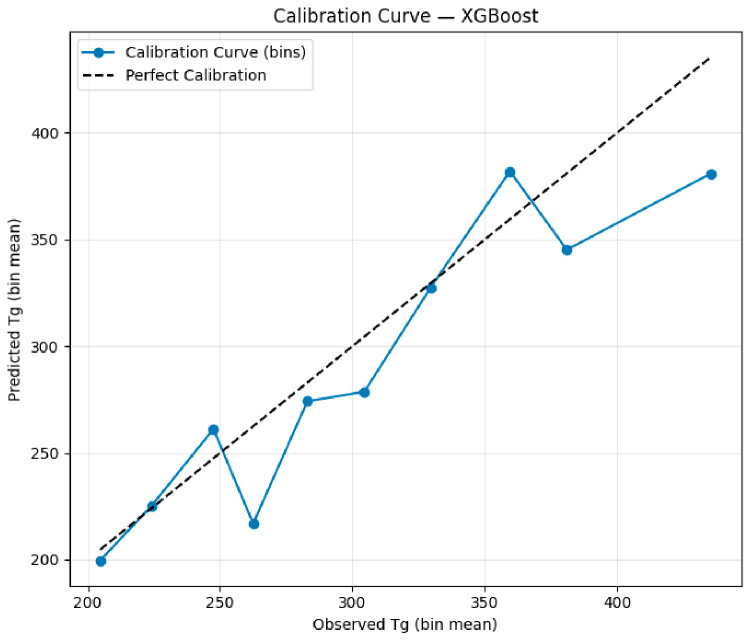
Calibration curve for the final model. The dashed line indicates perfect calibration (predicted mean equals observed mean).

Based on the screening results, XGBoost model was selected as the final regression model because it provided the best cross-validated predictivity and a competitive external test performance. The observed versus predicted plot (Figure 9) indicates that the model captures the global T_g_ trend, while the calibration curve (Figure 10) is used to verify the absence of strong systematic bias across the T_g_ range. Figure 9 shows the observed versus predicted glass transition temperatures (T_g_) obtained with the final model selected. Overall, the predictions follow the expected trend and cluster around the 1:1 reference line, indicating that the model captures the dominant structure–property signal across the investigated T_g_ range. In the low-to-intermediate T_g_ region, the agreement is generally close to the identity line, supporting the suitability of the selected descriptor set and learning strategy for practical screening purposes. A systematic pattern is also observed at the upper end of the T_g_ range, where the model tends to underpredict very high T_g_ values, a behavior consistent with a mild “regression-to-the-mean” effect commonly found in data-driven polymer property models. This tendency is typically associated with (i) reduced sample density at extreme T_g_ values, (ii) higher structural heterogeneity among high-T_g_ polymers, and (iii) limited representation of the corresponding descriptor space.

Figure 10 shows that the calibration curve for the model provides an additional assessment of prediction reliability across the T_g_ range by comparing the mean predicted T_g_ to the mean observed T_g_ within binned intervals. Overall, the curve follows the perfect-calibration line with reasonable agreement, indicating that the model is not strongly biased over most of the investigated domain. Local deviations from the identity line are observed in some intermediate bins, which is expected when calibration is computed from a limited number of samples per bin and when the chemical space is heterogeneous. This behavior would be improved over a broader database or on a mostly homogenic polymer dataset. In the upper T_g_ region, the curve suggests a tendency toward conservative predictions (i.e., reduced sensitivity at the extremes), consistent with the mild compression observed in the observed–predicted analysis. Despite these bin-level fluctuations, the calibration profile supports the use of the model for engineering screening, particularly within the T_g_ ranges that are more densely represented in the dataset.

**Figure 11:**
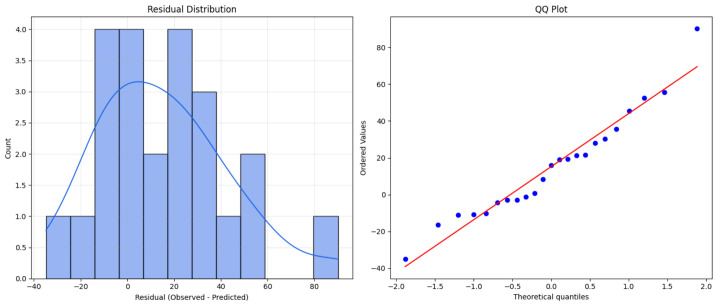
Residual distribution (observed–predicted T_g_) and Q–Q plot for the final model.

The residual analysis (Figure 11) provides further insight into model adequacy. The residual distribution (observed–predicted) is centered close to zero, indicating no severe global bias; however, a mild positive skewness is observed, with a longer right tail. This behavior suggests that a subset of samples, typically those located at the upper T_g_ end or at sparsely represented regions of the descriptor space, tend to be underpredicted by the model. The Q–Q plot supports this interpretation: residuals follow the reference line reasonably well in the central quantiles, while noticeable departures occur at the distribution tails, particularly at the upper tail. Such tail deviations are common in polymer QSPR datasets due to chemical-space heterogeneity, limited sample size, and the presence of structurally distinct compounds. Importantly, these diagnostics indicate that prediction errors are largely controlled for the majority of samples, while larger deviations are concentrated in a limited number of extreme cases, which should be considered when interpreting model uncertainty in high-T_g_ regimes.

**Figure 12:**
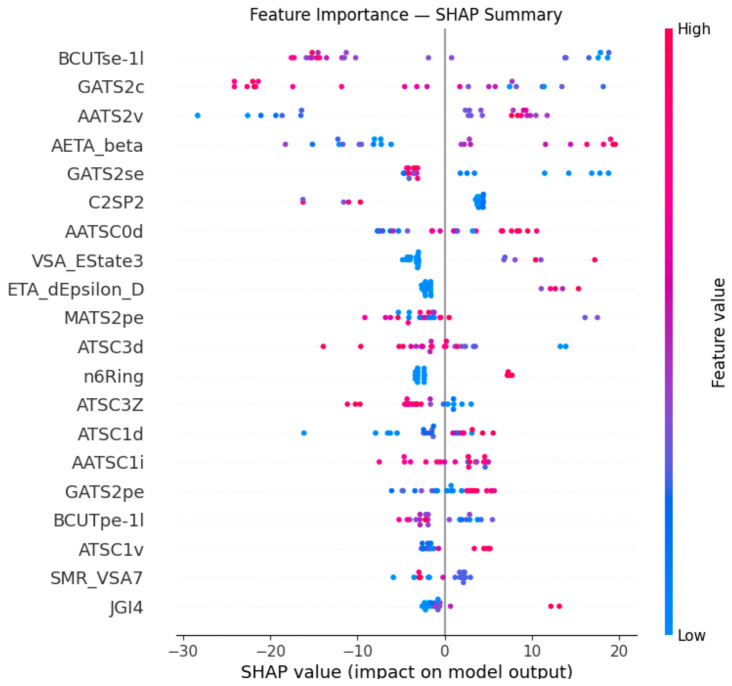
SHAP summary plot for the final XGBoost model. Each point represents a polymer; color indicates low-to-high feature values, and the horizontal position indicates the direction and magnitude of impact on predicted T_g_.

To enhance interpretability and support an engineering-oriented structure–property discussion, SHAP (SHapley Additive exPlanations) values were computed for the final model (Figure 12). The SHAP summary plot ranks descriptors according to their average absolute contribution to the predicted T_g_ and simultaneously indicates how low versus high descriptor values shift the model output.

Overall, the most influential variables are predominantly graph-based (2D) descriptors associated with molecular connectivity patterns (e.g., autocorrelation-type descriptors such as ATS2*), topological/shape-related indices (TSC* family), and ring-related terms. This attribution profile is consistent with the expected physicochemical determinants of the glass transition: polymer repeat units with higher structural rigidity, constrained connectivity, and increased ring/unsaturation content typically exhibit reduced segmental mobility and, therefore, higher T_g_. Importantly, SHAP does not imply causality; however, it provides a transparent attribution map that helps identify which structural motifs are driving the model’s predictions within the investigated chemical space.

**Figure 13:**
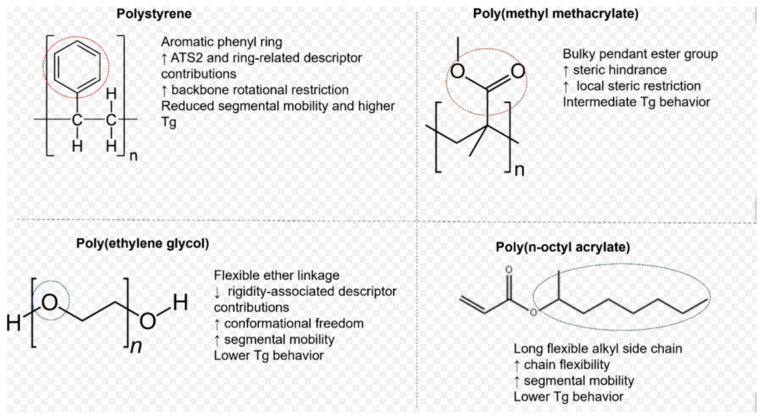
Representative polymer repeated units illustrating the relationship between dominant SHAP descriptor contributions and structural motifs associated with T_g_ behavior.

To enhance interpretability and support an engineering-oriented structure–property discussion, SHAP (SHapley Additive exPlanations) values were computed for the final XGBoost model (Figure 12). The SHAP summary plot ranks descriptors according to their average absolute contribution to the predicted T_g_ while simultaneously indicating how low versus high descriptor values shift the model output. Representative polymer repeats units associated with dominant descriptor trends are illustrated in Figure 13. Overall, the most influential variables are predominantly graph-based (2D) descriptors associated with molecular connectivity patterns, atomic-property autocorrelation, topological complexity, and cyclic/aromatic structural motifs. Among these, descriptors from the ATS2 family (e.g., AATS2v and ATS2*) encode autocorrelation relationships between atomic properties distributed across the molecular graph at short topological distances, reflecting how steric and electronic effects propagate through the repeat unit. Similarly, GATS2 descriptors are related to Geary autocorrelation functions and capture local topological heterogeneity and connectivity organization.

Polymers containing aromatic rings and rigid cyclic fragments, such as polystyrene-derived structures and phenylene-containing repeat units (Figure 13), generally exhibited higher contributions from ring-related and autocorrelation descriptors. These structural motifs restrict rotational freedom along the polymer backbone, increase local rigidity, and reduce segmental mobility, which is consistent with elevated T_g_ behavior. In contrast, polymers containing flexible ether linkages or long aliphatic side chains, such as poly(ethylene glycol)-type systems and long-chain alkyl acrylates (Figure 13), tended to present lower contributions from rigidity-associated descriptors and increased flexibility-related topological patterns. Such architectures favor conformational freedom and chain mobility, typically resulting in lower T_g_ values.

Descriptors associated with molecular topology and graph complexity, including ATSC*, BCUT, and ring-count-related terms (e.g., n6Ring), also contributed significantly to model predictions. Higher values for these descriptors were frequently associated with densely connected repeat units, aromaticity, and sterically constrained structures, all of which are classical physicochemical factors known to increase T_g_. Methacrylate-based polymers (Figure 13) presented intermediate behavior, where bulky pendant groups increase local steric hindrance and partially restrict chain mobility without reaching the rigidity levels observed in highly aromatic systems. This intermediate structural organization is reflected by moderate SHAP contributions from autocorrelation and topological descriptors.

Importantly, SHAP does not imply direct physicochemical causality; however, it provides a transparent attribution framework that helps identify which molecular connectivity patterns and structural motifs most strongly influence T_g_ predictions within the investigated chemical space.

## Figures and Tables

**Table 1:** Validation metrics for the training and test set using GA-LDA model.

	Training Set	Test Set
Number of compounds in the test	82	35
Accuracy	59.7561	54.2857
Precision	58.5366	56.25
Sensitivity	60	50
Specificity	59.5238	58.8235
F-measure	0.5926	0.5294
MCC	0.1952	0.0885
True Positive (train)	24	9
False Positive (train)	17	7
True Negative (train)	25	10
False Negative (train)	16	9

**Table 2:** Best models generated by the generation/iteration of the GA-LDA models.

Model Number	Descriptor 1	Descriptor 2	Descriptor 3	Fitness Score	Wilk’s Lambda
1	E2u	TDB10u	RDF25i	−0.6946	0.8473
2	E2s	TDB10e	RDF25i	−0.6961	0.8480
3	E2u	TDB10e	RDF65u	−0.7346	0.8673
4	E2u	TDB10e	RDF25u	−0.7456	0.8728
5	E2u	TDB10s	RDF25i	−0.7472	0.8736

**Table 3:** Comparative predictive performance of the evaluated regression models for T_g_ prediction.

Model	Q^2^_cv	RMSECV (K)	R^2^_Test	RMSE_Test (K)	MAE_Test (K)	MAE_%_Test
XGBoost	0.612	50.302	0.825	31.515	23.400	7.141
PLS	0.621	49.705	0.769	36.188	27.872	9.138
Random Forest	0.550	54.154	0.693	41.690	29.705	8.826
SVR	0.072	77.772	0.101	71.372	57.730	19.663

## Data Availability

The data is available upon request to the authors.

## Supplementary Materials

The following supporting information can be downloaded at: https://www.mdpi.com/article/10.3390/polym18111335/s1, Table S1: Set of 117 polymers used in this study; Table S2: Validation metrics for the training set using GA-LDA model; Table S3: Validation metrics for the test set using GA-LDA model; Table S4: Wilks’ Lambda values; Table S5: GA-LDA training model results for Model 1 (from Table 2); Table S6: GA-LDA test model results for Model 1 (from Table 2); Table S7: Standard Approach Training for the polymers used in this study; Table S8: Standard Approach Test for the polymers used in this study.
