# Supplementary material for: Use of Three-Dimensional Molecular Descriptors to Predict the Glass Transition Temperature of Polymers
Source: Polymers (Basel). 2026 May 28;18(11):1335. doi: 10.3390/polym18111335 (PMC13259346; doi:10.3390/polym18111335)
Supplement: Supplementary file 1 [file polymers-18-01335-s001.zip › polymers-4296042-supplementary/Supplementary Material_File S1.pdf]

Table S1. Set of 117 polymers used in this study.

Polymer	Acronym	Chemical structure	SMILES	Tg (K)	Source
Polystyrene	*PS*	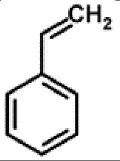	C=Cc1ccccc1	373	Polymer database
poly(methyl methacrylate)	*PMMA*	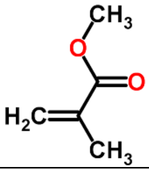	COC(=O)C(=C)C	378	Polymer database
poly(ethyl methacrylate)	*PEMA*	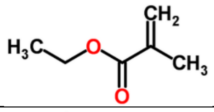	CCOC(=O)C(=C)C	338	Polymer database
Poly(2-methylstyrene)	*PMS*	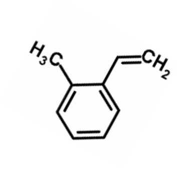	C=Cc1ccccc1C	409	Polymer database
Poly(4-tert-butylstyrene)	*PtBS*	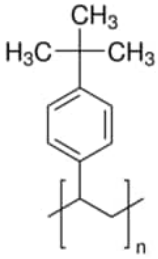	CC(C)(C)c1ccc(C=C)cc1	413	Sigma Aldritch
Poly(4-chlorostyrene)	*PCLS*	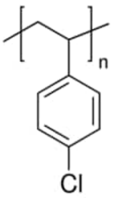	Clc1ccc(C=C)cc1	379	Sigma Aldritch
Poly(4-bromostyrene)	*PMB*	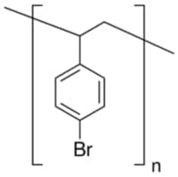	C=Cc1ccc(cc1)Br	411	Polymer database
Poly(benzyl methacrylate)	*PBzMA*	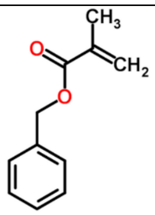	O=C(C(=C)C)OCc1ccccc1	327	Polymer database
Poly(cyclohexyl methacrylate)	*PCHMA*	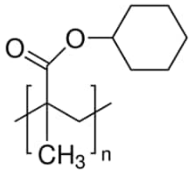	CC(=C)C(=O)OC1CCCCC1	377	Sigma Aldritch

Poly(butyl methacrylate)	*PnBMA*	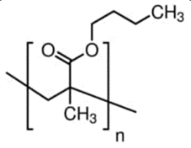	CCCCOC(=O)C(C)=C	303	Sigma Aldritch
Poly(ethylene)	*PE*	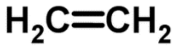	C=C	149	Polymer database
Poly(acrylamide)	*PAM*	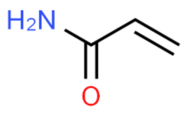	OC(=N)C=C	438	Polymer database
Poly[(N-tert-butylaminocarbonyl)ethylene]		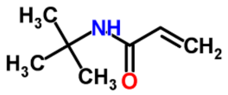	C=CC(=O)NC(C)(C)C	401	Polymer database
Poly(N-isopropyl acrylamide)		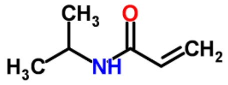	C=CC(=O)NC(C)C	403	Polymer database
Poly[(N-octylaminocarbonyl)ethylene]		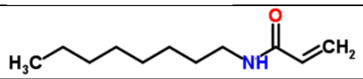	CCCCCCCCNC(=O)C=C	220	Polymer database
Poly[(N-phenylaminocarbonyl)ethylene]		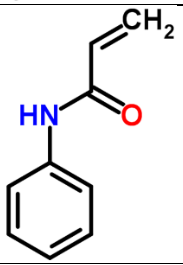	C=CC(=O)Nc1ccccc1		Polymer database
Poly[(N-sec-butylaminocarbonyl)ethylene]		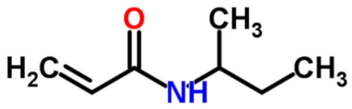	C=CC(=O)NC(C)CC	390	Polymer database
Poly(acrylic acid)	*PAA*	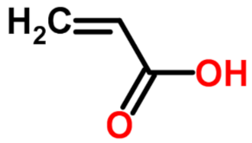	OC(=O)C=C	374	Polymer database
Poly(benzyl acrylate)		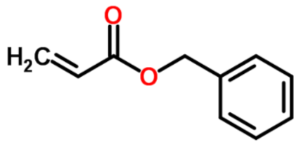	C=CC(=O)OCc1ccccc1	282	Polymer database
Poly(butyl acrylate)		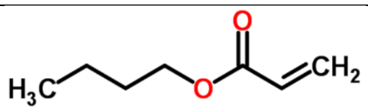	CCCCOC(=O)C=C	220	Polymer database
Poly(4-chlorophenyl acrylate)		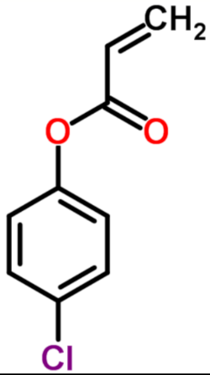	C=CC(=O)Oc1ccc(cc1)Cl	331	Polymer database

Poly(2-cyanoethyl acrylate)		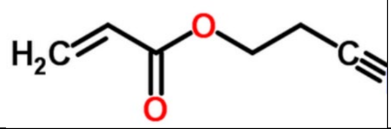	C=CC(=O)OCCC#N	277	Polymer database
Poly(cyanomethyl acrylate)		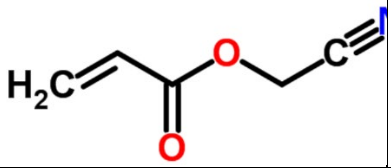	C=CC(=O)OCC#N	296	Polymer database
Poly(cyclohexyl acrylate)		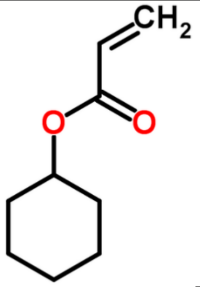	C=CC(=O)OC1CCCCC1	289	Polymer database
Poly(ethyl acrylate)		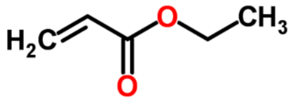	CCOC(=O)C=C	250	Polymer database
Poly(2-ethylhexyl acrylate)		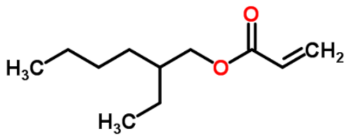	CCCCC(COC(=O)C=C)CC	221	Polymer database
Poly(hexyl acrylate)		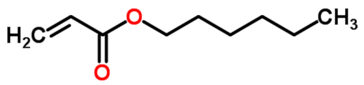	CCCCCCOC(=O)C=C	215	Polymer database
Poly(isobutyl acrylate)		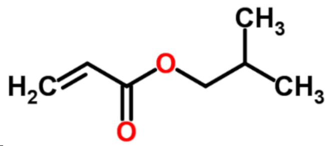	C=CC(=O)OCC(C)C	240	Polymer database
Poly(isopropyl acrylate)		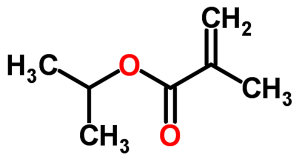	CC(C)OC(=O)C=C	271	Polymer database
Poly(methyl acrylate)		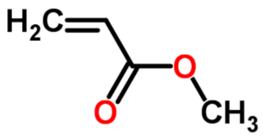	COC(=O)C=C	280	Polymer database
Poly(n-octyl acrylate)		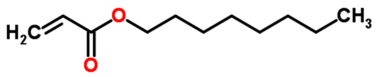	CCCCCCCCOC(=O)C=C	208	Polymer database
Poly(propyl acrylate)		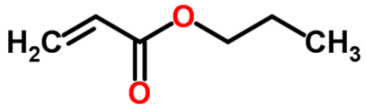	CCCOC(=O)C=C	231	Polymer database
Poly(sec-butyl acrylate)		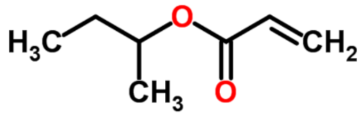	CC(OC(=O)C=C)CC	253	Polymer database

Poly(octadecyl acrylate)		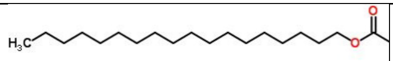	CCCCCCCCCCCCCCCC<br>CCCCCCCCCCOC(=O)C=C	311	Polymer database
Poly(tert-butyl acrylate)		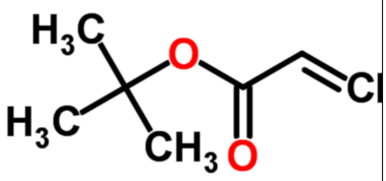	C=CC(=O)OC(C)(C)C	311	Polymer database
Poly(2,2,3,3-tetrafluoropropyl acrylate)		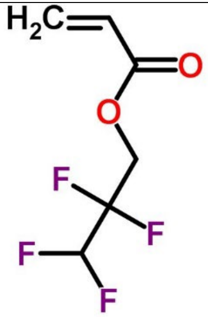	FC(F)C(F)(F)COC(=O)C(=C)	249	Polymer database
Poly(acrylonitrile)		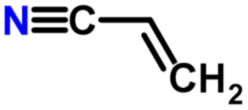	C=CC#N	383	Polymer database
Poly(methacrylonitrile)		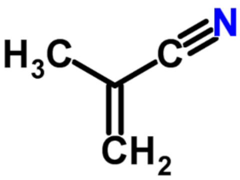	CC(=C)C#N	388	Polymer database
Poly(butylene adipate)		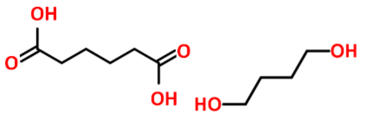	OC(=O)CCCCC(=O)O.OCCCCO	202	Polymer database
Poly(ethylene adipate)		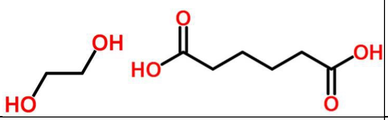	OC(=O)CCCCC(=O)O.OCCO	230	Polymer database
Poly(1,3-propylene adipate)		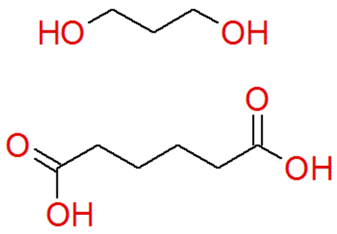	OC(=O)CCCCC(=O)O.OCCCO	215	Polymer database
Poly(ethyl ethylene		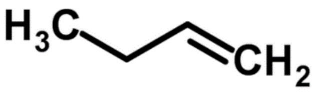	CCC=C	245	Polymer database
Poly(butyl ethylene)		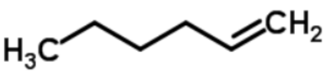	CCCCC=C	223	Polymer database
Poly(cyclohexylethylene)		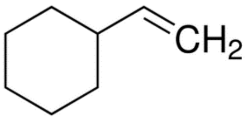	C=CC1CCCCC1	400	Polymer database
Poly(heptylethylene)		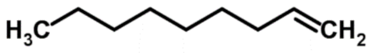	CCCCCCCC=C	225	Polymer database

Poly(hexylethylene)		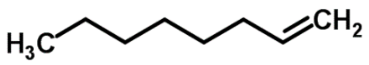	CCCCCCC=C	218	Polymer database
Poly(isobutene)		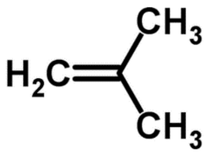	CC(=C)C	202	Polymer database
Poly(isobutylethylene)		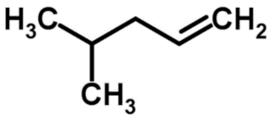	C=CCC(C)C	302	Polymer database
Poly(isopropylethylene		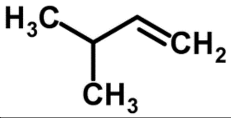	C=CC(C)C	323	Polymer database
Poly(1-ethyl-1-methylethylene)		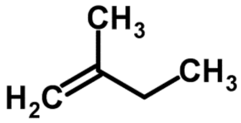	C=C(C)CC	268	Polymer database
Poly(octylethylene)		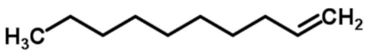	CCCCCCCCC=C	232	Polymer database
Poly(pentylethylene)		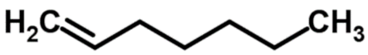	CCCCCC=C	231	Polymer database
Poly(propylene)	*PP*	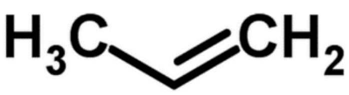	CC=C	264	Polymer database
Poly(propylethylene)		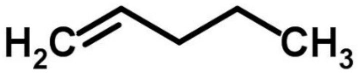	CCCC=C	238	Polymer database
Poly(tert-butylethylene)		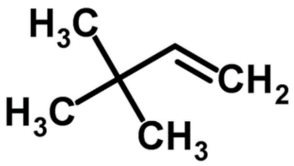	C=CC(C)(C)C	337	Polymer database
Polyamide 3	*PA3*	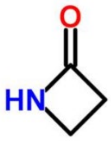	C1CNC1=O	384	Polymer database
Polyamide 6	*PA6*	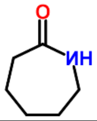	O=C1CCCCCN1	325	Polymer database
Polyamide 8	*PA8*	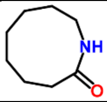	O=C1CCCCCCCN1	324	Polymer database
Polyamide 11	*PA11*	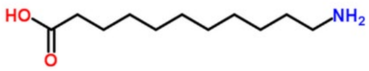	NCCCCCCCCCCC(=O)O	315<br>344	Polymer database
Polyamide 12	*PA12*	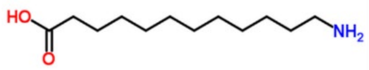	NCCCCCCCCCCCC(=O)O	313	Polymer database
Polyamide 4-6	*PA4-6*	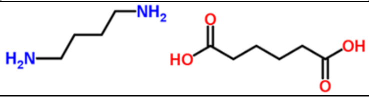	OC(=O)CCCCC(=O)O.NCCCCN	353	Polymer database

Polyamide 6-6	*PA6-6*	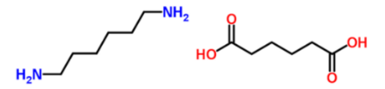	NCCCCCCN.OC(=O)CCCCC(=O)O	324	Polymer database
Polyamide 6-9	*PA6-9*	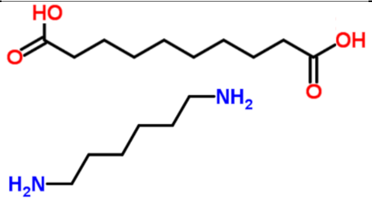	OC(=O)CCCCCCCC(=O)O.NCCCCCCN	332	Polymer database
Polyamide 6-10	*PA6-10*	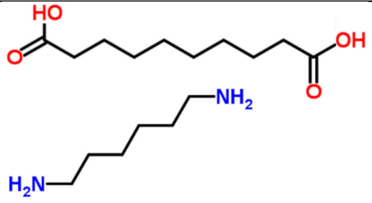	OC(=O)CCCCCCCCC(=O)O.NCCCCCCN	323	Polymer database
Polyamide 6-12	*PA6-12*	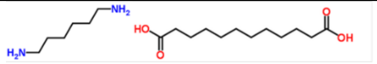	NCCCCCCN.OC(=O)CCCCCCCCCCC(=O)O	319	Polymer database
Polyamide 10-10	*PA10-10*	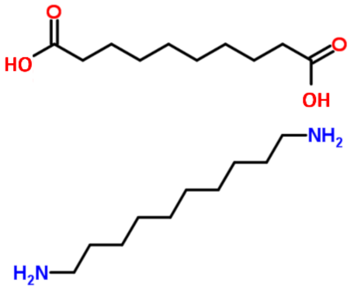	OC(=O)CCCCCCCCC(=O)O.NCCCCCCCCCCN	323	Polymer database
Poly(iminoisophthaloyliminohexamethylene	*PA 6I*	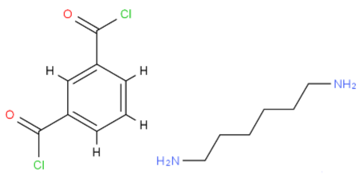	NCCCCCCN.ClC(=O)c1cc(ccc1)C(=O)Cl	395	Polymer database
Poly(hexamethylene teraphthalamide)	*PA 6T*	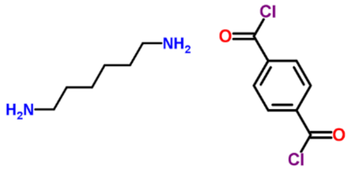	NCCCCCCN.ClC(=O)c1ccc(cc1)C(=O)Cl	412	Polymer database
Poly(p-phenylene terephthalamide)	*PPTA*	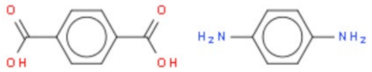	Nc1ccc(cc1)N.ClC(=O)c1ccc(cc1)C(=O)Cl	600	Polymer database
Poly(nonamethylene teraphthalamide)	*PP 9T*	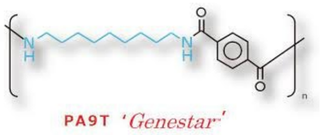	NCCCCCCCCCN.ClC(=O)c1ccc(cc1)C(=O)Cl	394	Polymer database
Poly(m-phenylene terephthalamide)	*MPDI*	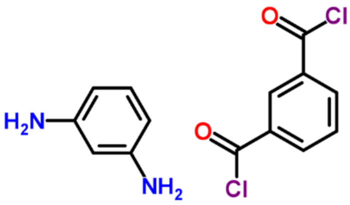	Nc1cc(ccc1)N.ClC(=O)c1cc(ccc1)C(=O)Cl	537	Polymer database
Poly(4,4'-pentamethylenedibenzoic anhydride)		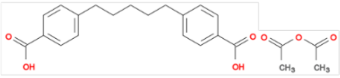	OC(=O)c1ccc(cc1)CCCCCc1ccc(cc1)C(=O)O.CC(=O)OC(=O)C	320	Polymer database

Poly(p-tetramethylenedibenzoic anhydride)		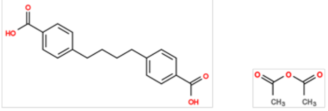	OC(=O)c1ccc(cc1)CCCCc1ccc(cc1)C(=O)O.CC(=O)OC(=O)C	316	Polymer database
Poly(sebacic anhydride)		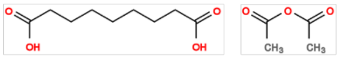	OC(=O)CCCCCCCCC(=O)O.CC(=O)OC(=O)C		Polymer database
Poly(azelaic anhydride)		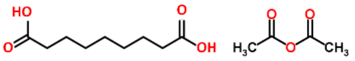	OC(=O)CCCCCCCC(=O)O.CC(=O)OC(=O)C		Polymer database
Poly(1,2-butadiene)		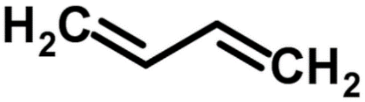	C=CC=C	261	Polymer database
Poly(1,4-butadiene)	*PBD*	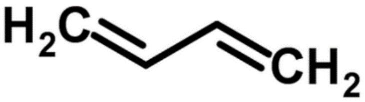	C=CC=C	171	Polymer database
Poly(1-pentenylene)	*PCP, PPT, CPR*	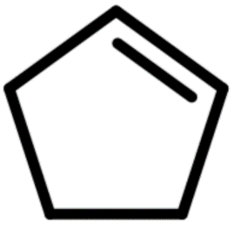	C1CC=CC1	178	Polymer database
Poly(1-ethyl-1,4-butadiene)		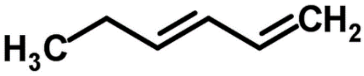	CCC=CC=C	197	Polymer database
Polyisoprene	*IR*	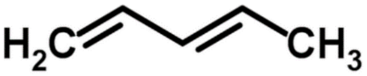	CC=CC=C	204	Polymer database
Poly(1,4-pentadiene)		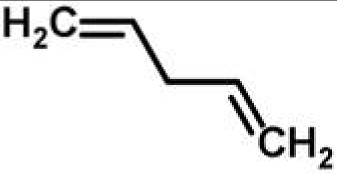	C=CCC=C		Polymer database
Poly(dimethyl fumarate)		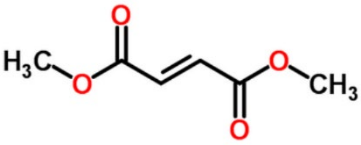	COC(=O)/C=C/C(=O)OC	373	Polymer database
Poly(dibutyl fumarate)		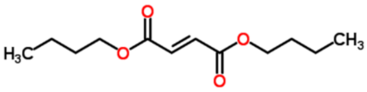	CCCCOC(=O)/C=C/C(=O)OCCCC		Polymer database
Poly(diethyl fumarate)		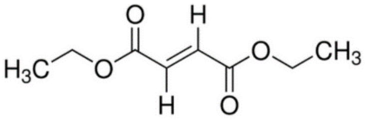	CCOC(=O)/C=C/C(=O)OCC	284	Polymer database
Poly(dipropyl fumarate)		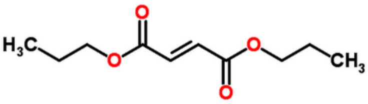	CCCOC(=O)/C=C/C(=O)OCCC	268	Polymer database
Poly(chlorotrifluoroethylene)	*PCTFE, PTFCE*	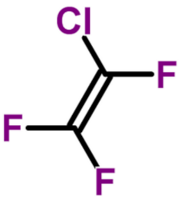	FC(=C(F)F)Cl	360	Polymer database

Poly(tetrafluoroethylene)	*PTFE*	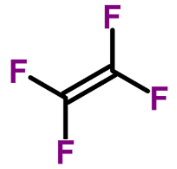	FC(=C(F)F)F	392<br>211	Polymer database
Poly(vinyl bromide)		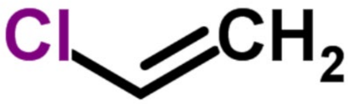	BrC=C	373	Polymer database
Poly(vinyl chloride)	*PVC*	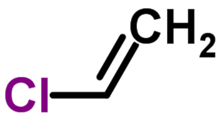	ClC=C	350<br>375	Polymer database
Poly(vinyl fluoride)	*PVF*	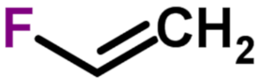	FC=C	253<br>326	Polymer database
Poly(vinylidene chloride)	*PVDC*	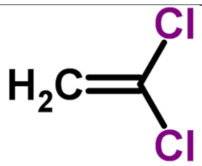	ClC(=C)Cl	256<br>284	Polymer database
Poly(vinylidene fluoride)	*PVDF*	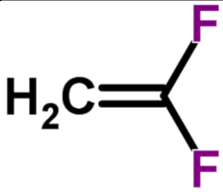	FC(=C)F	242	Polymer database
Poly(Bisphenol A-co-epichlorohydrin)		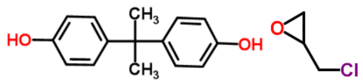	CC(C)(c1ccc(cc1)O)c2ccc(cc2)O.<br>C1C(O1)CCl	372	Polymer database
Poly(Bisphenol F-co-epichlorohydrin)	*DGEBF*	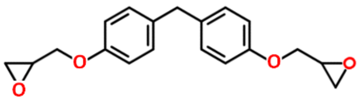	O1C<br>C1COc1ccc(cc1)Cc1ccc(cc1)OC<br>C1O C1	353	Polymer database
Poly(bisphenol-A diglycidyl ether-alt - ethylenediamine),		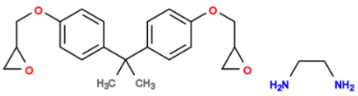	CC(c1ccc(cc1)OCC1OC1<br>)(c1ccc(cc1)OCC1OC1)C.NCCN	410	Polymer database
Poly(bisphenol-A diglycidyl ether-alt-1,6-diamino hexane)		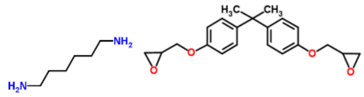	CC(c1ccc(cc1)OCC1OC1)(c1ccc(c<br>c1)OCC1OC1)C.NCCCCCCN	386	Polymer database
Poly(bisphenol A isophthalate)		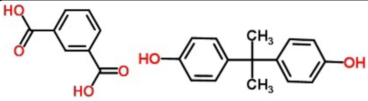	OC(=O)c1cc(ccc1)C(=O)O.Oc1cc<br>c(cc1)C(C)(C)c2ccc(cc2)O	462	Polymer database
Poly(Bisphenol A terephthalate)		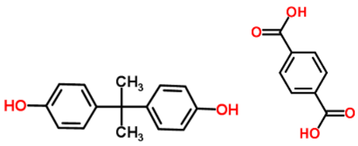	OC(=O)c1ccc(cc1)C(=O)<br>O.Oc1ccc(cc1)C(C)(C)c2ccc(cc2)<br>O	472	Polymer database
Poly(tetramethylene isophthalate)		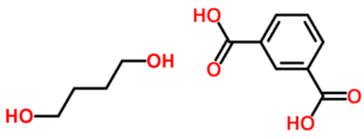	OC(=O)c1cccc(c1)C(=O)O.OCCC<br>CO	297	Polymer database

Poly(1,4-butylene sebacate)		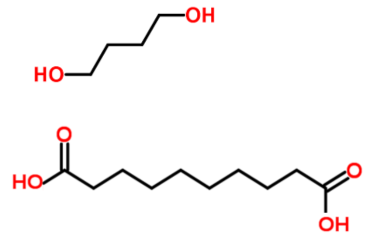	OC(=O)CCCCCCCCC(=O)O.OCCCCO	214	Polymer database
Poly(1,4-butylene succinate)		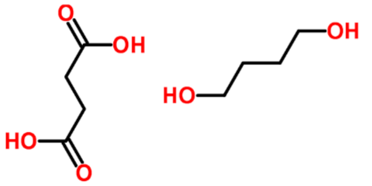	OC(=O)CCC(=O)O.OCCCCO	238	Polymer database
Poly[(tetramethylene terephthalate),	*PBT*	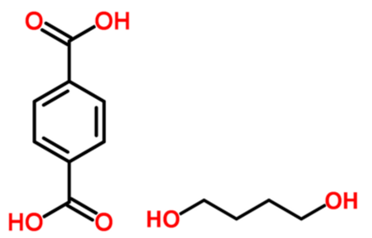	OC(=O)c1ccc(cc1)C(=O)O.OCCCCO	313	Polymer database
Poly(ethylene sebacate)		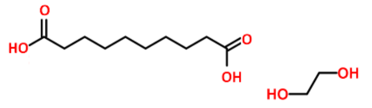	OC(=O)CCCCCCCCC(=O)O.OCCO	244	Polymer database
Poly(ethylene succinate)		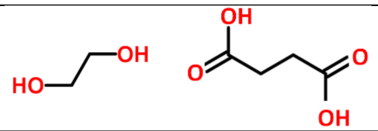	OC(=O)CCC(=O)O.OCCO	267	Polymer database
Poly(caprolactone)	*PCL*	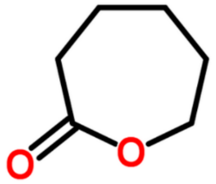	O=C1CCCCCO1	207	Polymer database
Poly(cyclohexanedimethylene terephthalate)	*PCT*	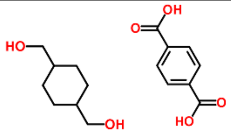	OC(=O)c1ccc(cc1)C(=O)O.OCC1CCC(CC1)CO	364	Polymer database
Poly(ethylene adipate)		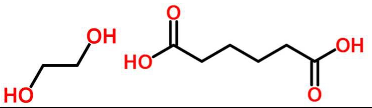	OC(=O)CCCCC(=O)O.OCCO	230	Polymer database
Poly(ethylene isophthalate)		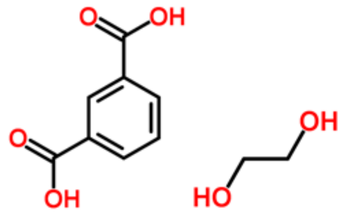	OC(=O)c1cccc(c1)C(=O)O.OCCO	324	Polymer database
Poly(ethylene 2,6-naphthalate)	*PEN*	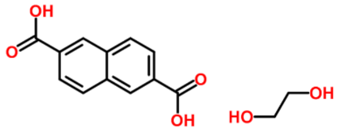	OC(=O)c1ccc2c(c1)ccc(c2)C(=O)O.OCCO	392	Polymer database

Poly(ethylene phthalate)		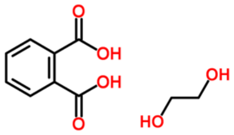	OC(=O)c1ccccc1C(=O)O.OCCO	297	Polymer database
Poly(ethylene terephthalate)	*PET*	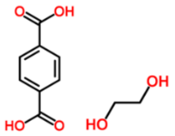	OC(=O)c1ccc(cc1)C(=O)O.OCCO	344	Polymer database
Polyglycolide	*PGA*	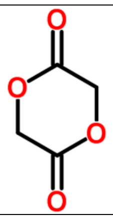	O=C1OCC(=O)OC1	312	Polymer database
Poly(1,6-hexylene sebacate)		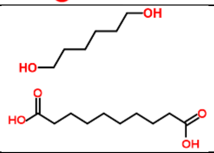	OC(=O)CCCCCCCCC(=O)O.OCCCCCCO	213	Polymer database
Poly(1,6-hexylene succinate)		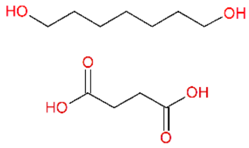	OC(=O)CCC(=O)O.OCCCCCCO	220	Polymer database
Poly(3-hydroxybutyrate)	*P3HB, PHB*	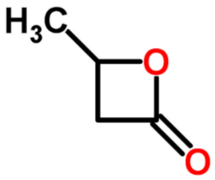	CC1CC(=O)O1	276	Polymer database
Poly(4-hydroxybutyrate)	*P4HB*	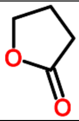	O=C1CCCO1	221	Polymer database
Polylactic acid,	*PLA*	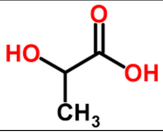	OC(=O)C(O)C	332	Polymer database
Poly(1,3-propylene adipate)		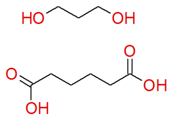	OC(=O)CCCCC(=O)O.OCCCO	215	Polymer database
Poly(1,3-propylene succinate)		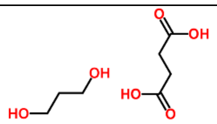	OC(=O)CCC(=O)O.OCCCO	232	Polymer database
Poly(trimethylene terephthalate)	*PTT*	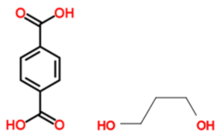	OC(=O)c1ccc(cc1)C(=O)O.OCCCO	326	Polymer database
Polyacetal	*POM*	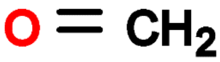	C=O	211	Polymer database
Poly(3-butoxypropylene oxide)		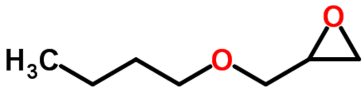	CCCCOCC1CO1	194	Polymer database

Poly(epichlorohydrin)		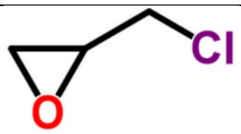	ClCC1CO1	253	Polymer database
Poly(ethylene glycol)	*PEO, POE, PEG*	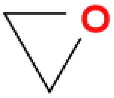	O1CC1	225	Polymer database
Poly(hexamethylene glycol)		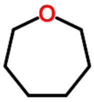	C1CCCCOC1	204	Polymer database
Poly(3-methoxypropylene oxide)		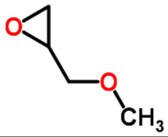	COCC1CO1	211	Polymer database
Poly[oxy(hexyloxymethyl)ethylene]		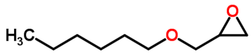	CCCCCCOCC1CO1	189	Polymer database
Poly(methylene oxide-co-ethylene oxide)		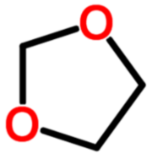	C1COCO1	209	Polymer database
Poly(propylene glycol)	*PPG*	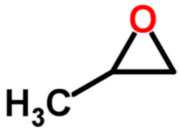	CC1CO1	205	Polymer database
Poly(tetrahydrofuran)	*PTMO, PTMEG*	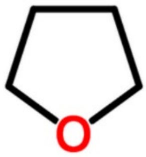	C1CCCO1	189	Polymer database
Poly(trimethylene glycol)		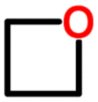	C1CCO1	197	Polymer database
Poly[1,1-bis(chloromethyl)trimethylene oxide]		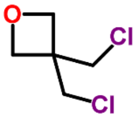	C1C(CO1)(CCl)CCl	272	Polymer database
Poly(ether ketone)	*PEK*	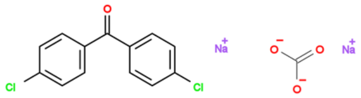	[O-]C(=O)[O-].[Na+].[Na+].O=C(c1ccc(cc1)Cl)c1ccc(cc1)Cl	431	Polymer database
Poly(ether ether ketone)	*PEKK*	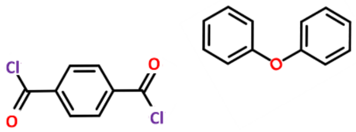	c1cc(ccc1C(=O)Cl)C(=O)Cl.c1ccc(cc1)Oc2ccccc2	437	Polymer database
Poly(ether ether ketone)	*PEEK*	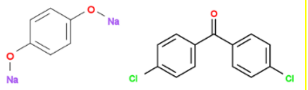	[Na]Oc1ccc(cc1)O[Na].O=C(c1ccc(cc1)Cl)c1ccc(cc1)Cl	421	Polymer database
Poly(ethyleneketone)		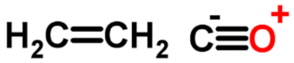	C=C.[C-]#[O+]	291	Polymer database
Poly(propyleneketone)	*PPK*	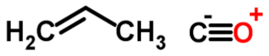	CC=C.[C-]#[O+]	312	Polymer database

Poly(ether ether sulfone)	*PEES*	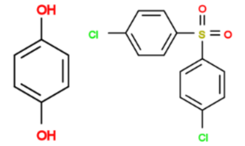	Oc1ccc(cc1)O.Clc1ccc(cc1)S(=O)(=O)c1ccc(cc1)Cl	479	Polymer database
Poly(ethersulfone)	*PES*	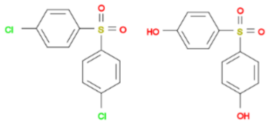	Clc1ccc(cc1)S(=O)(=O)c1ccc(cc1)Cl.Oc1ccc(cc1)S(=O)(=O)c1ccc(cc1)O	490	Polymer database
Poly(phenylsulfone)	*PPSU*<br>*PPSF*	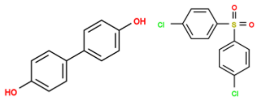	Oc1ccc(cc1)c1ccc(cc1)O.Clc1ccc(cc1)S(=O)(=O)c1ccc(cc1)Cl	482	Polymer database
Bisphenol A Polysulfone	*PSU*<br>*PSF*	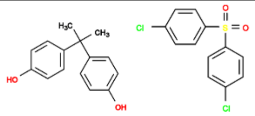	CC(c1ccc(cc1)O)(c1ccc(cc1)O)C.Clc1ccc(cc1)S(=O)(=O)c1ccc(cc1)Cl	459	Polymer database
Polychloroprene		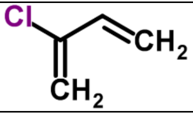	C=CC(=C)Cl	237	Polymer database
Poly(1-bromo-1-butenylene)		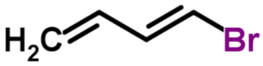	BrC=CC=C	233	Polymer database
Poly(2-chlorostyrene)		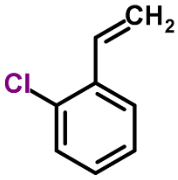	C=Cc1ccccc1Cl	395	Polymer database
Poly(3-chlorostyrene)		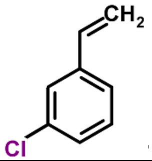	C=Cc1cccc(c1)Cl	363	Polymer database
Poly(4-chlorostyrene)		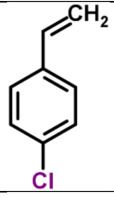	C=Cc1ccc(cc1)Cl	398	Polymer database
Poly(2,5-difluorostyrene)		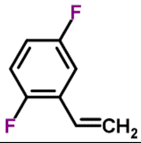	C=Cc1cc(F)ccc1F	374	Polymer database
Poly(4-fluorostyrene)		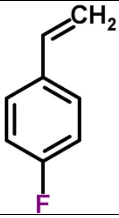	C=Cc1ccc(cc1)F	376	Polymer database
Poly(2-hydroxyethyl acrylate)	*PHEA*	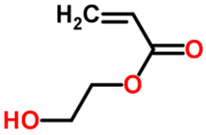	C=CC(=O)OCCO	259	Polymer database

Poly(2-hydroxyethyl methacrylate)	*PHEMA*	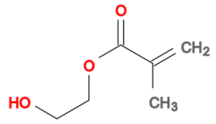	CC(=C)C(=O)OCCO	358<br>393	Polymer database
Poly(2-hydroxypropyl methacrylate)	*PHPMA*	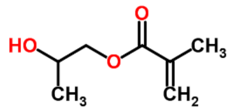	CC(COC(=O)C(=C)C)O	349	Polymer database
Poly[(diethylene glycol)-alt-(1,6-hexamethylene diisocyanate)],	*PEG 100 - HMDI*	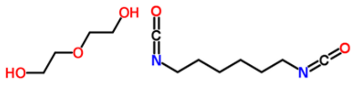	OCCOCCO.O=C=NCCCCCCN=C=O	281	Polymer database
Poly[(tetraethylene glycol)-alt-(1,6-hexamethylene diisocyanate)],	*PEG 200 - HMDI*	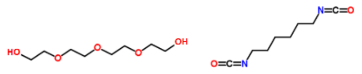	OCCOCCOCCOCCO.O=C=NCCCCCCN=C=O	258	Polymer database
Poly[(1,4-butanediol)-alt-(4,4'-diphenylmethane diisocyanate)]	*MDI-BD*	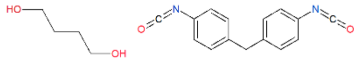	OCCCCO.O=C=Nc1ccc(cc1)Cc1ccc(cc1)N=C=O	363	Polymer database
Poly{(ethylene glycol)-alt-[bis(4-isocyanatophenyl)methane]}	*EG - MDI*	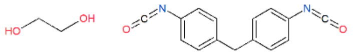	OCCO.O=C=Nc1ccc(cc1)Cc1ccc(cc1)N=C=O	412	Polymer database
Poly[(tetramethylene ether glycol 1000)-alt-(4,4'-diphenylmethane diisocyanate)],	*PTMG 1000 - MDI*	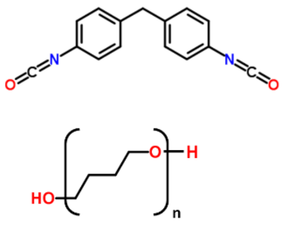	(OCCCC)14O.O=C=Nc1ccc(cc1)Cc1ccc(cc1)N=C=O	223	Polymer database
Poly(bisphenol A isophthalate)		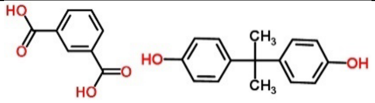	OC(=O)c1cc(ccc1)C(=O)O.Oc1ccc(cc1)C(C)(C)c2ccc(cc2)O	462	Polymer database
Poly(tetramethylene isophthalate)		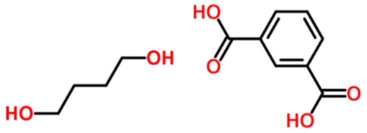	OC(=O)c1cccc(c1)C(=O)O.OCCCCO	297	Polymer database
Poly(ethylene isophthalate)		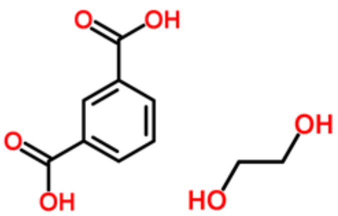	OC(=O)c1cccc(c1)C(=O)O.OCCO	324	Polymer database
Poly(dimethyl itaconate)		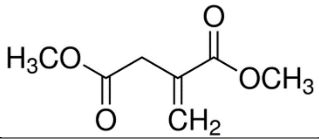	COC(=O)CC(=C)C(=O)OC	373	Polymer database
Poly[di(n-propyl) itaconate]		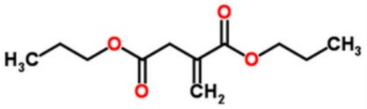	CCCOC(=O)CC(=C)C(=O)OCCC	306	Polymer database
Poly[di(n-butyl) itaconate		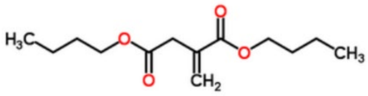	CCCCOC(=O)CC(=C)C(=O)OCCCC	285	Polymer database
Poly[di(n-hexyl) itaconate]		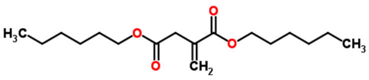	CCCCCCOC(=O)CC(=C)C(=O)OCCCCCC	255	Polymer database

Poly(N-acetylmethacrylamide)			CC(=O)NC(=O)C(=C)C		Polymer database
Poly(N-benzyl methacrylamide)		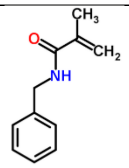	O=C(C(=C)C)NCc1ccccc1		Polymer da<br>Polymer database<br>tabase
Polymethacrylamide	*PMAAM*	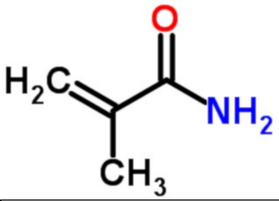	CC(=C)C(=O)N	486,5	Polymer database
Poly(N-tert-butylmethacrylamide)		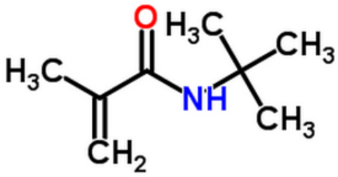	CC(=C)C(=O)NC(C)(C)C	433	Polymer database
Poly(p-phenylene)	*PPP*	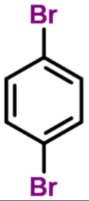	Brc1ccc(cc1)Br	538	Polymer database
Poly(p-phenylene vinylene)	*PPV*	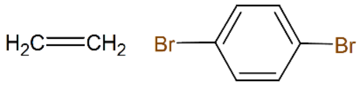	Brc1ccc(cc1)Br.C=C	353	Polymer database
Poly(p-xylene)		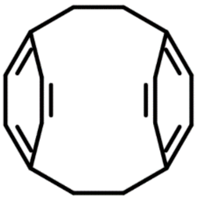	C1Cc2ccc(cc2)CCc2ccc1cc2	343	Polymer database
Poly(2-chloro-p-xylylene)		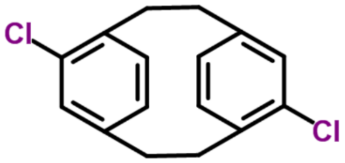	Clc1cc2CCc3ccc(CCc1cc2)cc3Cl	358	Polymer database
Poly(2,6-dimethyl-p-phenylene oxide)	*PPO*<br>*PPE*	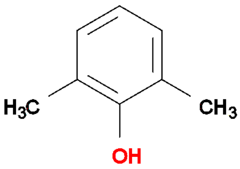	Oc1c(C)cccc1(C)	483	Polymer database
Poly(2,6-diphenyl-p-phenylene oxide)		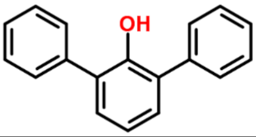	Oc1c(cccc1c1ccccc1)c1ccccc1	497	Polymer database
Poly(p-phenylene oxide)	*PPO, PPE*	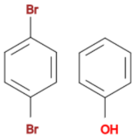	Brc1ccc(Br)cc1.Oc1ccccc1	361	Polymer database

Poly(ether ether sulfone)	*PEES*	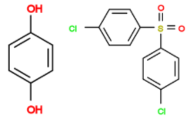	Oc1ccc(cc1)O.Clc1ccc(cc1)S(=O)(=O)c1ccc(cc1)Cl	479	Polymer database
Poly(ethersulfone)	*PES*	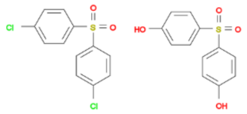	Clc1ccc(cc1)S(=O)(=O)c1ccc(cc1)Cl.Oc1ccc(cc1)S(=O)(=O)c1ccc(cc1)O	490	Polymer database
Poly(phenylsulfone)	*PPSU*<br>*PPSF*	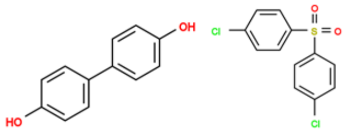	Oc1ccc(cc1)c1ccc(cc1)O.Clc1ccc(cc1)S(=O)(=O)c1ccc(cc1)Cl	482	Polymer database
Bisphenol A Polysulfone	*PSU*<br>*PSF*	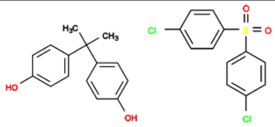	CC(c1ccc(cc1)O)(c1ccc(cc1)O)C.Clc1ccc(cc1)S(=O)(=O)c1ccc(cc1)Cl	459	Polymer database
Poly(1,4-phenylene sulfide)	*PPS*	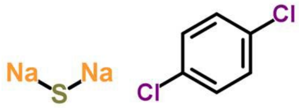	Clc1ccc(cc1)Cl.[Na]S[Na]	363	Polymer database
Poly(ethylene sulfide)	*TR*	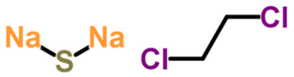	ClCCCl.[Na]S[Na]	223	Polymer database
Poly(propylene sulfide)		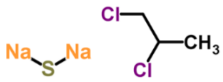	ClCC(C)Cl.[Na]S[Na]	228	Polymer database
Poly(1,4-butylene sebacate)		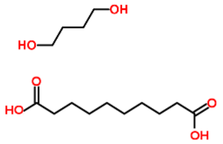	OC(=O)CCCCCCCCC(=O)O.OCCCCO	214	Polymer database
Poly(ethylene sebacate)		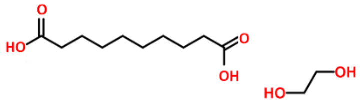	OC(=O)CCCCCCCCC(=O)O.OCCO	244	Polymer database
Poly(1,6-hexylene sebacate)		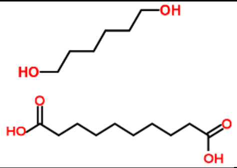	OC(=O)CCCCCCCCC(=O)O.OCCCCCCO	213	Polymer database
Poly(diethylsiloxane)	*PDES*	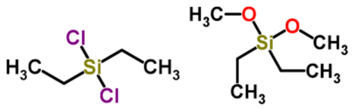	CC[Si](CC)(Cl)Cl, CC[Si](CC)(OC)OC	137	Polymer database
Poly(dimethylsiloxane)	*PDMS*	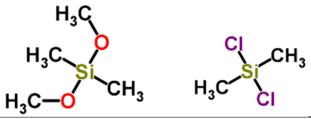	C[Si](C)(Cl)Cl, CO[Si](C)(C)OC	149	Polymer database
Poly(methylphenylsiloxane)	*PMPS*	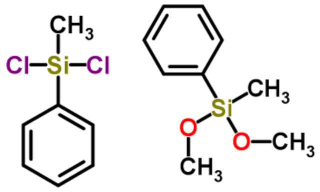	C[Si](c1ccccc1)(Cl)Cl, CO[Si](C)(c1ccccc1)OC	245	Polymer database
Poly(1,4-butylene succinate)		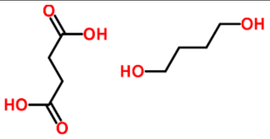	OC(=O)CCC(=O)O.OCCCCO	238	Polymer database

Poly(ethylene succinate		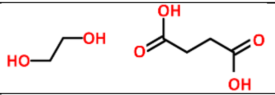	OC(=O)CCC(=O)O.OCCO	267	Polymer database
Poly(1,6-hexylene succinate)		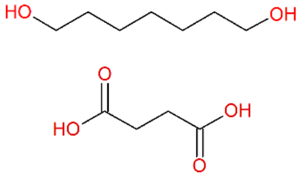	OC(=O)CCC(=O)O.OCCCCCCO	220	Polymer database
Poly(1,3-propylene succinate)		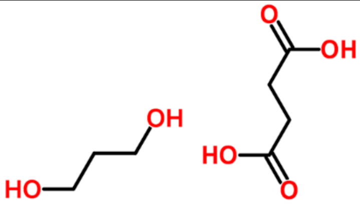	OC(=O)CCC(=O)O.OCCCO	232	Polymer database
Poly(Bisphenol A terephthalate)		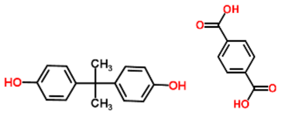	OC(=O)c1ccc(cc1)C(=O)O.Oc1ccc(cc1)C(C)(C)c2ccc(cc2)O	472	Polymer database
Poly[(tetramethylene terephthalate)	*PBT*	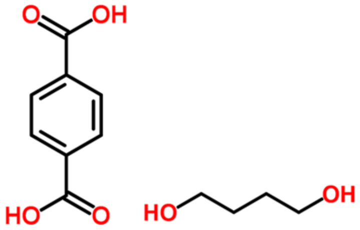	OC(=O)c1ccc(cc1)C(=O)O.OCCCCO	313	Polymer database
Poly(cyclohexanedimethylene terephthalate)	*PCT*	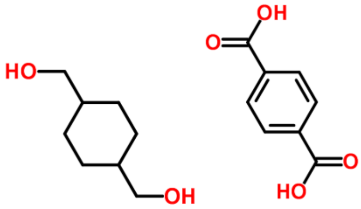	OC(=O)c1ccc(cc1)C(=O)O.OCC1CCC(CC1)CO	364	Polymer database
Poly(ethylene terephthalate)	*PET*	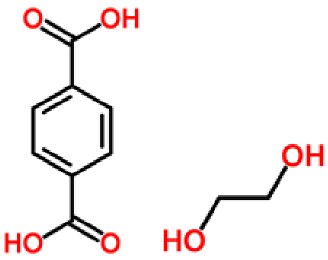	OC(=O)c1ccc(cc1)C(=O)O.OCCO	344	Polymer database
Poly(trimethylene terephthalate)	*PTT*	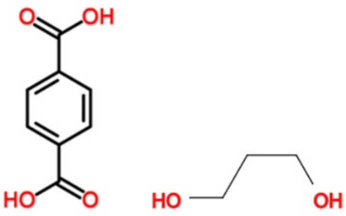	OC(=O)c1ccc(cc1)C(=O)O.OCCCO	326	Polymer database
Poly(vinyl alcohol)	*PVOH, PVA*	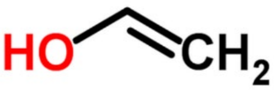	C{-}(OC(=O)C)C{n+}	353	Polymer database
Poly(4-hydroxystyrene)	*PVP*	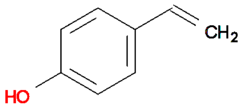	C=Cc1ccc(cc1)O	430<br>473	Polymer database

Poly(vinyl butyral)	*PVB*	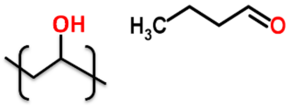	C{-}C(O){n+}.O=CCCC	344	Polymer database
Poly(vinyl formal)	*PVF*	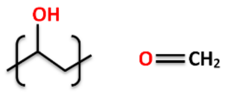	C{-}C(O){n+}.O=C	325	Polymer database
Poly(vinyl acetate)	*PVAc* *PVA*	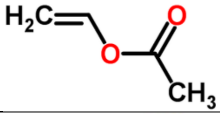	CC(=O)OC=C	307	Polymer database
Poly(vinylbenzoate)		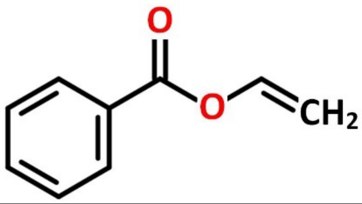	C=COC(=O)c1ccccc1	344	Polymer database
Poly(vinyl butyrate)	*PVB*	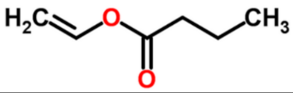	CCCC(=O)OC=C		Polymer database
Poly(vinyl caproate)		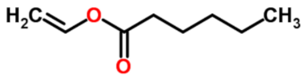	CCCCCC(=O)OC=C		Polymer database
Poly(vinyl formate)		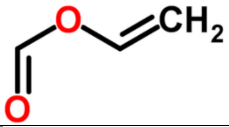	C=COC=O	307	Polymer database
Poly(vinyl propionate		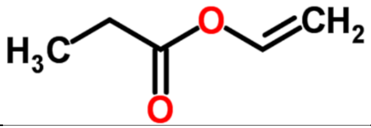	C=COC(=O)CC	283	Polymer database
Poly(vinyl sterate)		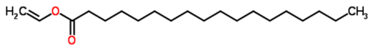	CCCCCCCCCCCCCCCCCC(=O)OC=C	318	Polymer database
Poly(vinyl valerate)		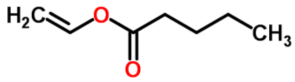	CCCCC(=O)OC=C		Polymer database
Poly(vinyl ethyl ketone)		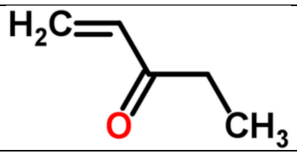	CCC(=O)C=C	213	Polymer database
Poly(vinyl methyl ketone)		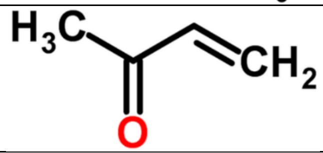	CC(=O)C=C	301	Polymer database
Poly(methyl isopropenyl ketone)		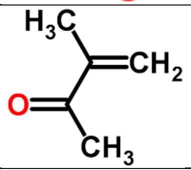	CC(=O)C(C)=C	370	Polymer database
Poly(vinyl phenyl ketone)		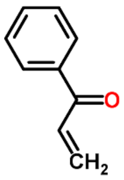	C=CC(=O)c1ccccc1	323	Polymer database

Poly(butyl vinyl thioether)		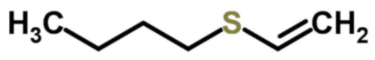	CCCCSC=C	253	Polymer database
Poly(ethyl vinyl thioether)		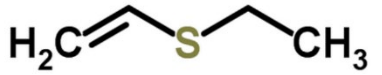	CCSC=C	266	Polymer database
Poly(methyl vinyl thioether)		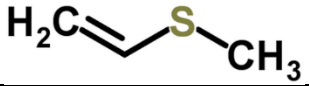	CSC=C	272	Polymer database
Poly(vinyl phenyl sulfide)		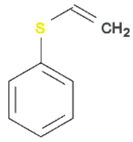	C=CSc1ccccc1	386	Polymer database
Poly(propyl vinyl thioether)		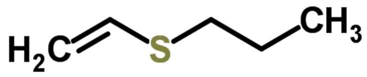	CCCSC=C		Polymer database

poly(acrilomide), para-substituted polystyrenes, POLYACRYLATES (POLYPROPENOATES), Polynitrile, Polyesters, POLYAKLENES (POLYOLEFINS), POLYAMIDES (POLYLACTAMS), POLYANHYDRIDES, POLYDIENES, POLYFUMARATES (POLYBUTENEDIOATES), POLYHALOOLEFINS, POLYFLUOROOLEFINS, POLYCHLOROOLEFINS, POLYEPOXIDES (POLYHYDROXYETHERS) EPOXY & PHENOXY POLYMERS , POLYESTERS, POLYGLYCOLS (POLYETHERS) (MONOMERS: GLYCIDYL ETHERS & CYCLIC ETHERS), POLYKETONES POLYETHERKETONES, POLYETHERSULFONES (POLYSULFONE), POLYFUMARATES (POLYBUTENEDIOATES), POLYGLYCOLS (POLYETHERS) (MONOMERS: GLYCIDYL ETHERS & CYCLIC ETHERS), POLYHALODIENES CHLORINATED AND BROMINATED DIENES, POLYHALOSTYRENES CHLORINATED AND FLUORINATED STYRENES, POLYHYDROXY(METH)ACRYLATES, POLYURETHANES, POLYISOPHTHALATE, POLYITACONATES, (POLYMETHYLENE SUCCINATES), POLYMETHACRYLAMIDES, POLYPHENYLENES (POLYAROMATICS), POLYPHENYLETHERS POLYPHENYLENEOXIDE, POLYETHERSULFONES (POLYSULFONE), POLYSULFIDES POLYTHIOETHERS, POLYSEBACATES, POLYSILOXANES (SILICONES), POLYSUCCINATES, POLYTEREPHTHALATES, POLYVINYL ALCOHOLS & DERIVATIVES, POLYVINYL ESTERS, POLYVINYL KETONES, POLYVINYL SULFIDES (POLYVINYL THIOETHERS),
