# Supplementary material for: Use of Three-Dimensional Molecular Descriptors to Predict the Glass Transition Temperature of Polymers
Source: Polymers (Basel). 2026 May 28;18(11):1335. doi: 10.3390/polym18111335 (PMC13259346; doi:10.3390/polym18111335)
Supplement: Supplementary file 1 [file polymers-18-01335-s001.zip › polymers-4296042-supplementary/Supplementary MAterial_File S4.pdf]

Table S2. Validation metrics for the training set using GA-LDA model.

| CompID | ClassID(Obs.) | ClassID(Pred.) | PosteriorProb.(P) | PosteriorProb.(N) |
|---|---|---|---|---|
| PMMA | P | P | 0.543015 | 0.456985 |
| PEMA | P | P | 0.632098 | 0.367902 |
| PMS | P | N | 0.463270 | 0.536730 |
| PtBS | N | P | 0.596033 | 0.403967 |
| PCLS | N | P | 0.563438 | 0.436562 |
| PMB | P | P | 0.574773 | 0.425227 |
| PBzMA | N | N | 0.068212 | 0.931788 |
| PCHMA | N | N | 0.493282 | 0.506718 |
| PmBHA | P | P | 0.726452 | 0.273548 |
| PE | P | P | 0.940237 | 0.059763 |
| PAM | N | P | 0.560719 | 0.439281 |
| Poly (N-tert-butylaminocarbonyl | P | P | 0.669455 | 0.330545 |
| Poly(N-isopropyl acrylamide) | P | P | 0.571228 | 0.428772 |
| Poly (N-octylaminocarbonyl)ethy | N | N | 0.163046 | 0.836954 |
| Poly (N-sec-butylaminocarbonyl) | P | P | 0.700845 | 0.299155 |
| PAA | P | P | 0.632553 | 0.367447 |
| Poly(benzyl acrylate) | N | N | 0.182146 | 0.817854 |
| Poly(butyl acrylate) | P | P | 0.645815 | 0.354185 |

| | | | | |
|---|---|---|---|---|
| Poly(4-chlorophenyl acrylate) | N | P | 0.656536 | 0.343464 |
| Poly(2-cyanoethyl acrylate) | N | N | 0.403233 | 0.596767 |
| Poly(cyanomethyl acrylate) | P | P | 0.554941 | 0.445059 |
| Poly(cyclohexyl acrylate) | P | P | 0.536367 | 0.463633 |
| Poly(ethyl acrylate) | N | P | 0.560362 | 0.439638 |
| Poly(2-ethylhexyl acrylate) | P | N | 0.312426 | 0.687574 |
| Poly(hexyl acrylate) | N | N | 0.134519 | 0.865481 |
| Poly(isobutyl acrylate) | P | P | 0.627811 | 0.372189 |
| Poly(isopropyl acrylate) | N | P | 0.611764 | 0.388236 |
| Poly(methyl acrylate) | P | P | 0.651104 | 0.348896 |
| Poly(n-octyl acrylate) | N | N | 0.171259 | 0.828741 |
| Poly(propyl acrylate) | N | P | 0.634371 | 0.365629 |
| Poly(sec-butyl acrylate) | N | P | 0.699534 | 0.300466 |
| Poly(octadecyl acrylate) | P | P | 0.938433 | 0.061567 |
| Poly(tert-butyl acrylate) | P | P | 0.677232 | 0.322768 |

| | | | | |
|---|---|---|---|---|
| Poly(2.2.3.3-tetrafluoropropyl | N | N | 0.430243 | 0.569757 |
| Poly(acrylonitrile) | P | P | 0.642228 | 0.357772 |
| Poly(methacrylonitrile) | N | N | 0.350572 | 0.649428 |
| Poly(ethyl ethylene | P | N | 0.435768 | 0.564232 |
| Poly(butyl ethylene) | P | N | 0.362694 | 0.637306 |
| Poly(cyclohexylethylene) | N | N | 0.356999 | 0.643001 |
| Poly(heptylethylene) | N | N | 0.248275 | 0.751725 |
| Poly(hexylethylene) | P | N | 0.318969 | 0.681031 |
| Poly(isobutylethylene) | N | N | 0.449800 | 0.550200 |
| Poly(isopropyl ethylene | P | N | 0.429823 | 0.570177 |
| Poly(1-ethyl-1-methylethylene) | N | N | 0.043115 | 0.956885 |
| Poly(octylethylene) | P | N | 0.263138 | 0.736862 |
| Poly(pentylethylene) | N | N | 0.326458 | 0.673542 |
| Poly(propylene) | P | P | 0.621697 | 0.378303 |
| Poly(tert-butylethylene) | N | P | 0.502094 | 0.497906 |
| PA3 | P | N | 0.411443 | 0.588557 |

| | | | | |
|---|---|---|---|---|
| PA6 | P | N | 0.471596 | 0.528404 |
| PA8 | N | N | 0.411469 | 0.588531 |
| PA11 | N | N | 0.218598 | 0.781402 |
| PVDC | N | N | 0.326437 | 0.673563 |
| PVDF | N | N | 0.229211 | 0.770789 |
| PCL | P | N | 0.411454 | 0.588546 |
| PHB | P | P | 0.728138 | 0.271862 |
| POM | P | P | 0.940237 | 0.059763 |
| Poly(ethylene glycol) | N | N | 0.326439 | 0.673561 |
| Poly(3-methoxypropylene oxide) | P | P | 0.621699 | 0.378301 |
| Poly oxy(hexyloxy methyl)ethylen | N | N | 0.486783 | 0.513217 |
| Poly(methylene oxide-co-ethylen | P | N | 0.382224 | 0.617776 |
| Poly 1.1-bis(chloromethyl)trime | N | P | 0.577771 | 0.422229 |
| Poly(2-chlorostyrene) | N | P | 0.532564 | 0.467436 |
| Poly(2.5-difluorostyrene) | P | P | 0.517343 | 0.482657 |
| Poly(4-fluorostyrene) | N | P | 0.517341 | 0.482659 |
| Poly(2-hydroxyethyl acrylate) | N | N | 0.396747 | 0.603253 |

| | | | | |
|---|---|---|---|---|
| Poly(2-hydroxyethyl methacrylat | P | N | 0.411449 | 0.588551 |
| Poly di(npropyl) itaconate | N | P | 0.532520 | 0.467480 |
| Poly di(nhexyl) itaconate | P | N | 0.486818 | 0.513182 |
| Polymethacryl amide | N | N | 0.456403 | 0.543597 |
| Poly(Ntertbuty lmethacrylami de | N | N | 0.382241 | 0.617759 |
| Poly(p-phenylene) | P | N | 0.396740 | 0.603260 |
| Poly(p-xylene) | N | N | 0.441326 | 0.558674 |
| Poly(2-chloro-p-xylylene) | P | P | 0.562825 | 0.437175 |
| Poly(2.6-diphenyl-p-phenylene o | P | N | 0.456360 | 0.543640 |
| Poly(4-hydroxystyren e) | N | P | 0.547730 | 0.452270 |
| Poly(vinyl propionate) | N | P | 0.502084 | 0.497916 |
| Poly(vinyl ethyl ketone) | P | N | 0.456404 | 0.543596 |
| Poly(methyl isopropenyl ketone) | N | P | 0.577768 | 0.422232 |
| Poly(ethyl vinyl thioether) | N | P | 0.532564 | 0.467436 |

| | | | | |
|---|---|---|---|---|
| Poly(vinyl phenyl sulfide) | P | P | 0.607240 | 0.392760 |
| Poly(propyl vinyl thioether) | N | N | 0.441305 | 0.558695 |

Table S3. Validation metrics for the test set using GA-LDA model.

| CompID | ClassID(Obs.) | ClassID(Pred.) | PosteriorProb.(P) | PosteriorProb.(N) |
|---|---|---|---|---|
| PS | N | P | 0.601043 | 0.398957 |
| Poly(isobutene) | N | N | 0.471116 | 0.528884 |
| Poly(propylethylene) | P | N | 0.411449 | 0.588551 |
| PA12 | P | N | 0.218602 | 0.781398 |
| Poly(1.2-butadiene) | N | N | 0.441299 | 0.558701 |
| PCP | N | N | 0.326446 | 0.673554 |
| Poly(vinyl bromide) | N | N | 0.426301 | 0.573699 |
| PVC | N | N | 0.367915 | 0.632085 |
| PVF | P | N | 0.240175 | 0.759825 |
| PGA | N | N | 0.154310 | 0.845690 |
| P4HB | N | N | 0.300182 | 0.699818 |
| PLA | P | P | 0.547726 | 0.452274 |
| Poly(3-butoxypropylene oxide) | N | P | 0.502098 | 0.497902 |
| Poly(epichlorohydrin) | N | P | 0.592584 | 0.407416 |

| | | | | |
|---|---|---|---|---|
| Poly(hexamethylene glycol) | P | N | 0.382238 | 0.617762 |
| PPG | P | P | 0.592582 | 0.407418 |
| Poly(tetrahydrofuran) | P | N | 0.326439 | 0.673561 |
| Poly(trimethylene glycol) | P | P | 0.728138 | 0.271862 |
| Polychloroprene | N | P | 0.577762 | 0.422238 |
| Poly(1-bromo-1-butenylene) | P | P | 0.940237 | 0.059763 |
| Poly(3-chlorostyrene) | N | P | 0.577764 | 0.422236 |
| Poly(4-chlorostyrene) | P | P | 0.517339 | 0.482661 |
| Poly(2-hydroxypropyl methacryla | N | N | 0.471596 | 0.528404 |
| Poly(dimethyl itaconate) | P | P | 0.562807 | 0.437193 |
| Poly di(nbutyl) itaconate | P | P | 0.502051 | 0.497949 |
| Poly(2.6-dimethyl-p-phenylene o | P | N | 0.486830 | 0.513170 |

| | | | | |
|---|---|---|---|---|
| Poly(vinyl acetate) | N | P | 0.562803 | 0.437197 |
| Poly(vinylbenzoate) | P | P | 0.532569 | 0.467431 |
| Poly(vinyl formate) | N | N | 0.426302 | 0.573698 |
| Poly(vinyl sterate) | P | N | 0.251520 | 0.748480 |
| Poly(vinyl methyl ketone) | N | P | 0.502085 | 0.497915 |
| Poly(vinyl phenyl ketone) | P | P | 0.577768 | 0.422232 |
| Poly(vinylpyrrolidone) | N | N | 0.367920 | 0.632080 |
| Poly(butyl vinyl thioether) | P | N | 0.396756 | 0.603244 |
| Poly(methyl vinyl thioether) | P | N | 0.471583 | 0.528417 |

Table S4. Wilks' Lambda values.

| MODEL TYPE | Wilks Lambda | MODEL TYPE | Wilks Lambda |
|---|---|---|---|
| ORIGINAL MODEL | 0.8473107385435035 | RANDOM MODEL26 | 0.9385437823554674 |
| RANDOM MODEL1 | 0.9724804848169114 | RANDOM MODEL27 | 0.9451827731158358 |
| RANDOM MODEL2 | 0.9791674256555506 | RANDOM MODEL28 | 0.9764386479355238 |
| RANDOM MODEL3 | 0.9949918094048382 | RANDOM MODEL29 | 0.9189575500797847 |
| RANDOM MODEL4 | 0.950502732520164 | RANDOM MODEL30 | 0.9671946929094901 |
| RANDOM MODEL5 | 0.9916734744021328 | RANDOM MODEL31 | 0.9777565351682173 |
| RANDOM MODEL6 | 0.9404579241809711 | RANDOM MODEL32 | 0.9900115518446453 |
| RANDOM MODEL7 | 0.9847926418536992 | RANDOM MODEL33 | 0.9667294176724204 |
| RANDOM MODEL8 | 0.9954606595674339 | RANDOM MODEL34 | 0.9393785530379563 |
| RANDOM MODEL9 | 0.9884855286531439 | RANDOM MODEL35 | 0.9469338794721999 |
| RANDOM MODEL10 | 0.988491356750838 | RANDOM MODEL36 | 0.95457672643199 |
| RANDOM MODEL11 | 0.9818294081200999 | RANDOM MODEL37 | 0.9677727925767463 |
| RANDOM MODEL12 | 0.9743702534966244 | RANDOM MODEL38 | 0.9449918873537934 |
| RANDOM MODEL13 | 0.977389285293009 | RANDOM MODEL39 | 0.9834872447854861 |
| RANDOM MODEL14 | 0.9547252603586497 | RANDOM MODEL40 | 0.9568737046166255 |
| RANDOM MODEL15 | 0.9620331768697995 | RANDOM MODEL41 | 0.9554502392658748 |
| RANDOM MODEL16 | 0.9565942902446422 | RANDOM MODEL42 | 0.9320861901768209 |
| RANDOM MODEL17 | 0.946700313669694 | RANDOM MODEL43 | 0.9463340568500274 |
| RANDOM MODEL18 | 0.9585159490583591 | RANDOM MODEL44 | 0.9846727885050415 |
| RANDOM MODEL19 | 0.9692580069844812 | RANDOM MODEL45 | 0.9517474156283141 |
| RANDOM MODEL20 | 0.999400598833617 | RANDOM MODEL46 | 0.954457737048728 |
| RANDOM MODEL21 | 0.9329109636468202 | RANDOM MODEL47 | 0.9270331297364106 |
| RANDOM MODEL22 | 0.9677658515128814 | RANDOM MODEL48 | 0.9721006265631685 |
| RANDOM MODEL23 | 0.9651255250015168 | RANDOM MODEL49 | 0.9718753605636224 |

| | | | |
|---|---|---|---|
| RANDOM MODEL24 | 0.9389181707855471 | RANDOM MODEL50 | 0.9289586396645185 |
| RANDOM MODEL25 | 0.9713856641003965 | | |
