# Supplementary material for: Use of Three-Dimensional Molecular Descriptors to Predict the Glass Transition Temperature of Polymers
Source: Polymers (Basel). 2026 May 28;18(11):1335. doi: 10.3390/polym18111335 (PMC13259346; doi:10.3390/polym18111335)
Supplement: Supplementary file 1 [file polymers-18-01335-s001.zip › polymers-4296042-supplementary/Supplementary MAterial_File S5.pdf]

Table S5. GA-LDA training model results for Model 1 (from Table 2).

Polymer	Response class	E2u	TDB10u	RDF25i
PMMA	P	0.427	0.0	5817.0
PEMA	P	0.4263	0.0	95059.0
PMS	P	0.5345	0.0	88251.0
PtBS	N	0.5128	0.0	187089.0
PCLS	N	0.4474	0.0	56549.0
PMB	P	0.4421	0.0	59944.0
PBzMA	N	0.4999	91965.0	15628.0
PCHMA	N	0.5777	0.0	182292.0
PmBHA	P	0.4376	0.0	218579.0
PE	P	0.0	0.0	0.0
PAM	N	0.4428	0.0	46967.0
Poly (N-tert-butylaminocarbonyl)	P	0.469	0.0	199171.0
Poly(N-isopropylacrylamide)	P	0.4917	0.0	130546.0
Poly (N-octylaminocarbonyl)ethyl	N	0.593	108214.0	497513.0
Poly (N-sec-butylaminocarbonyl)	P	0.4664	0.0	230936.0
PAA	P	0.3841	0.0	32451.0
Poly(benzylacrylate)	N	0.3692	99751.0	142047.0
Poly(butylacrylate)	P	0.4894	0.0	203951.0
Poly(4-chlorophenylacrylate)	N	0.4077	0.0	93371.0
Poly(2-cyanoethylacrylate)	N	0.522	0.0	9616.0
Poly(cyanomethylacrylate)	P	0.4526	0.0	55882.0
Poly(cyclohexylacrylate)	P	0.5394	0.0	167297.0
Poly(ethylacrylate)	N	0.417	0.0	8043.0

Poly(2-ethylhexylacrylate)	P	0.3988	104992.0	394136.0
Poly(hexylacrylate)	N	0.5382	104992.0	339856.0
Poly(isobutylacrylate)	P	0.4713	0.0	157829.0
Poly(isopropylacrylate)	N	0.4622	0.0	127549.0
Poly(methylacrylate)	P	0.3783	0.0	43543.0
Poly(n-octylacrylate)	N	0.5721	107433.0	475762.0
Poly(propylacrylate)	N	0.4604	0.0	148433.0
Poly(sec-butylacrylate)	N	0.4452	0.0	197713.0
Poly(octadecylacrylate)	P	0.6811	108555.0	1698911.0
Poly(tert-butylacrylate)	P	0.4168	0.0	129792.0
Poly(2,2,3,3-tetrafluoropropyl)	N	0.507	0.0	14408.0
Poly(acrylonitrile)	P	0.357	0.0	2188.0
Poly(methacrylonitrile)	N	0.555	0.0	3976.0
Poly(ethylethylene)	P	0.496	0.0	3474.0
Poly(butylethylene)	P	0.555	0.0	16913.0
Poly(cyclohexylethylene)	N	0.558	0.0	15345.0
Poly(heptylethylene)	N	0.612	10787.0	37299.0
Poly(hexylethylene)	P	0.596	0.0	30503.0
Poly(isobutylethylene)	N	0.494	0.0	14411.0
Poly(isopropylethylene)	P	0.504	0.0	9504.0
Poly(1-ethyl-1-methylethylene)	N	1.0	0.0	61185.0
Poly(octylethylene)	P	0.62	10.88	44.09
Poly(pentylethylene)	N	0.57	0.0	23.71
Poly(propylene)	P	0.37	0.0	3.66
Poly(tert-butylethylene)	N	0.45	0.0	16.92
PA3	P	0.51	0.0	3.91

PA6	P	0.47	0.0	15.69
PA8	N	0.51	0.0	30.2
PA11	N	0.66	10.73	62.74
PVDC	N	0.57	0.0	0.24
PVDF	N	0.65	0.0	2.83
PCL	P	0.51	0.0	15.46
PHB	P	0.29	0.0	4.48
POM	P	0.0	0.0	0.0
Poly(ethyleneglycol)	N	0.57	0.0	2.53
Poly(3-methoxypropylene oxide)	P	0.37	0.0	6.47
Polyoxy(hexyloxymethyl)ethylene	N	0.46	10.64	34.92
Poly(methylene oxide-co-ethylene)	P	0.53	0.0	2.89
Poly 1,1-bis(chloromethyl)ethylene	N	0.4	0.0	12.99
Poly(2-chlorostyrene)	N	0.43	0.0	6.28
Poly(2,5-difluorostyrene)	P	0.44	0.0	9.6
Poly(4-fluorostyrene)	N	0.44	0.0	8.0
Poly(2-hydroxyethylacrylate)	N	0.52	0.0	9.0
Poly(2-hydroxyethylmethacrylate)	P	0.51	0.0	10.5
Polydi(npropyl)itaconate	N	0.43	10.47	29.73
Polydi(nhexyl)itaconate	P	0.46	10.41	68.01
Polymethacrylamide	N	0.48	0.0	6.19
Poly(Ntertbutylmethacrylamide)	N	0.53	0.0	19.98
Poly(p-phenylene)	P	0.52	0.0	2.27
Poly(p-xylene)	N	0.49	0.0	31.4
Poly(2-chloro-p-xylylene)	P	0.41	0.0	27.83
Poly(2,6-diphenyl-p-phenylene)oxide	P	0.48	9.28	22.23

Poly(4-hydroxystyrene)	N	0.42	0.0	8.11
Poly(vinylpropionate)	N	0.45	0.0	7.1
Poly(vinylethylketone)	P	0.48	0.0	7.09
Poly(methylisopropenylketone)	N	0.4	0.0	9.59
Poly(ethylvinylthioether)	N	0.43	0.0	6.34
Poly(vinylphenylsulfide)	P	0.38	0.0	10.03
Poly(propylvinylthioether)	N	0.49	0.0	11.06

Table S6. GA-LDA test model results for Model 1 (from Table 2).

Polymer	Response class	E2u	TDB10u	RDF25i
PS	N	0.44	0.0	83362.0
Poly(isobutene)	N	0.475	0.0	7019.0
Poly(propylethylene)	P	0.51	0.0	10.21
PA12	P	0.66	10.76	69.54
Poly(1,2-butadiene)	N	0.49	0.0	5.35
PCP	N	0.57	0.0	10.58
Poly(vinylbromide)	N	0.5	0.0	1.69
PVC	N	0.54	0.0	2.6
PVF	P	0.64	0.0	1.92
PGA	N	0.73	0.0	6.53
P4HB	N	0.59	0.0	5.42
PLA	P	0.42	0.0	4.22
Poly(3-butoxypropyleneoxide)	N	0.45	0.0	21.33
Poly(epichlorohydrin)	N	0.39	0.0	5.87
Poly(hexamethyleneglycol)	P	0.53	0.0	16.92
PPG	P	0.39	0.0	3.87
Poly(tetrahydrofuran)	P	0.57	0.0	2.78
Poly(trimethyleneglycol)	P	0.29	0.0	5.05
Polychloroprene	N	0.4	0.0	3.96
Poly(1-bromo-1-butenylene)	P	0.0	0.0	0.0
Poly(3-chlorostyrene)	N	0.4	0.0	6.04
Poly(4-chlorostyrene)	P	0.44	0.0	6.21
Poly(2-hydroxypropylmethacrylate)	N	0.47	0.0	15.77

Poly(dimethylitaconate)	P	0.41	0.0	9.79
Poly di(nbutyl)itaconate	P	0.45	10.35	40.83
Poly(2,6-dimethyl-p-phenylene)oxide	P	0.46	0.0	13.9
Poly(vinylacetate)	N	0.41	0.0	5.22
Poly(vinylbenzoate)	P	0.43	0.0	10.96
Poly(vinylformate)	N	0.5	0.0	2.64
Poly(vinylsterate)	P	0.63	10.87	105.91
Poly(vinylmethylketone)	N	0.45	0.0	7.92
Poly(vinylphenyl ketone)	P	0.4	0.0	10.05
Poly(vinylpyrrolidone)	N	0.54	0.0	7.58
Poly(butylvinylthioether)	P	0.52	0.0	17.85
Poly(methylvinylthioether)	P	0.47	0.0	3.45

Table S7. Standard Approach Training for the polymers used in this study.

Polymer	E2u	TDB10u	RDF25i	Response class	Predicted Class	Status (AD)
PMMA	0.427	0.0	5817.0	P	P	In
PEMA	0.4263	0.0	95059.0	P	P	In
PMS	0.5345	0.0	88251.0	P	N	In
PtBS	0.5128	0.0	187089.0	N	P	In
PCLS	0.4474	0.0	56549.0	N	P	In
PMB	0.4421	0.0	59944.0	P	P	In
PBzMA	0.4999	91965.0	15628.0	N	N	In
PCHMA	0.5777	0.0	182292.0	N	N	In
PmBHA	0.4376	0.0	218579.0	P	P	In
PE	0.0	0.0	0.0	P	P	Outlier
PAM	0.4428	0.0	46967.0	N	P	In
Poly (N-tert-butylaminocarbonyl)	0.469	0.0	199171.0	P	P	In
Poly(N-isopropylacrylamide)	0.4917	0.0	130546.0	P	P	In
Poly (N-octylaminocarbonyl)ethylene	0.593	108214.0	497513.0	N	N	Outlier
Poly (N-sec-butylaminocarbonyl)	0.4664	0.0	230936.0	P	P	In
PAA	0.3841	0.0	32451.0	P	P	In
Poly(benzylacrylate)	0.3692	99751.0	142047.0	N	N	Outlier
Poly(butylacrylate)	0.4894	0.0	203951.0	P	P	In
Poly(4-chlorophenylacrylate)	0.4077	0.0	93371.0	N	P	In
Poly(2-cyanoethylacrylate)	0.522	0.0	9616.0	N	N	In
Poly(cyanomethylacrylate)	0.4526	0.0	55882.0	P	P	In
Poly(cyclohexylacrylate)	0.5394	0.0	167297.0	P	P	In
Poly(ethylacrylate)	0.417	0.0	8043.0	N	P	In
Poly(2-ethylhexylacrylate)	0.3988	104992.0	394136.0	P	N	Outlier
Poly(hexylacrylate)	0.5382	104992.0	339856.0	N	N	Outlier
Poly(isobutylacrylate)	0.4713	0.0	157829.0	P	P	In
Poly(isopropylacrylate)	0.4622	0.0	127549.0	N	P	In
Poly(methylacrylate)	0.3783	0.0	43543.0	P	P	In

Poly(n-octylacrylate)	0.5721	107433.0	475762.0	N	N	Outlier
Poly(propylacrylate)	0.4604	0.0	148433.0	N	P	In
Poly(sec-butylacrylate)	0.4452	0.0	197713.0	N	P	In
Poly(octadecylacrylate)	0.6811	108555.0	1698911.0	P	P	Outlier
Poly(tert-butylacrylate)	0.4168	0.0	129792.0	P	P	In
Poly(2,2,3,3-tetrafluoropropyl)	0.507	0.0	14408.0	N	N	In
Poly(acrylonitrile)	0.357	0.0	2188.0	P	P	In
Poly(methacrylonitrile)	0.555	0.0	3976.0	N	N	In
Poly(ethylethylene)	0.496	0.0	3474.0	P	N	In
Poly(butylethylene)	0.555	0.0	16913.0	P	N	In
Poly(cyclohexylethylene)	0.558	0.0	15345.0	N	N	In
Poly(heptylethylene)	0.612	10787.0	37299.0	N	N	In
Poly(hexylethylene)	0.596	0.0	30503.0	P	N	In
Poly(isobutylethylene)	0.494	0.0	14411.0	N	N	In
Poly(isopropylethylene	0.504	0.0	9504.0	P	N	In
Poly(1-ethyl-1-methylethylene)	1.0	0.0	61185.0	N	N	Outlier
Poly(octylethylene)	0.62	10.88	44.09	P	N	In
Poly(pentylethylene)	0.57	0.0	23.71	N	N	In
Poly(propylene)	0.37	0.0	3.66	P	P	In
Poly(tert-butylethylene)	0.45	0.0	16.92	N	P	In
PA3	0.51	0.0	3.91	P	N	In
PA6	0.47	0.0	15.69	P	N	In
PA8	0.51	0.0	30.2	N	N	In
PA11	0.66	10.73	62.74	N	N	In
PVDC	0.57	0.0	0.24	N	N	In
PVDF	0.65	0.0	2.83	N	N	In
PCL	0.51	0.0	15.46	P	N	In
PHB	0.29	0.0	4.48	P	P	In
POM	0.0	0.0	0.0	P	P	Outlier
Poly(ethyleneglycol)	0.57	0.0	2.53	N	N	In
Poly(3-methoxypropyleneoxide)	0.37	0.0	6.47	P	P	In
Poly oxy(hexyloxymethyl)ethylene	0.46	10.64	34.92	N	N	In

Poly(methylene oxide-co-ethylene)	0.53	0.0	2.89	P	N	In
Poly 1,1-bis(chloromethyl)trimethyl	0.4	0.0	12.99	N	P	In
Poly(2-chlorostyrene)	0.43	0.0	6.28	N	P	In
Poly(2,5-difluorostyrene)	0.44	0.0	9.6	P	P	In
Poly(4-fluorostyrene)	0.44	0.0	8.0	N	P	In
Poly(2-hydroxyethylacrylate)	0.52	0.0	9.0	N	N	In
Poly(2-hydroxyethylmethacrylate)	0.51	0.0	10.5	P	N	In
Polydi(npropyl)itaconate	0.43	10.47	29.73	N	P	In
Polydi(nhexyl)itaconate	0.46	10.41	68.01	P	N	In
Polymethacrylamide	0.48	0.0	6.19	N	N	In
Poly(Ntertbutylmethacrylamide)	0.53	0.0	19.98	N	N	In
Poly(p-phenylene)	0.52	0.0	2.27	P	N	In
Poly(p-xylene)	0.49	0.0	31.4	N	N	In
Poly(2-chloro-p-xylylene)	0.41	0.0	27.83	P	P	In
Poly(2,6-diphenyl-p-phenylene)oxide	0.48	9.28	22.23	P	N	In
Poly(4-hydroxystyrene)	0.42	0.0	8.11	N	P	In
Poly(vinylpropionate)	0.45	0.0	7.1	N	P	In
Poly(vinylethylketone)	0.48	0.0	7.09	P	N	In
Poly(methylisopropenylketone)	0.4	0.0	9.59	N	P	In
Poly(ethylvinylthioether)	0.43	0.0	6.34	N	P	In
Poly(vinylphenylsulfide)	0.38	0.0	10.03	P	P	In
Poly(propylvinylthioether)	0.49	0.0	11.06	N	N	In

Table S8. Standard Approach Test for the polymers used in this study.

Polymer	E2u	TDB10u	RDF25i	Response class	Predicted Class	Status (AD)
PS	0.44	0.0	83362.0	N	P	Inside-AD
Poly(isobutene)	0.475	0.0	7019.0	N	N	Inside-AD
Poly(propylethylene)	0.51	0.0	10.21	P	N	Inside-AD
PA12	0.66	10.76	69.54	P	N	Inside-AD
Poly(1,2-butadiene)	0.49	0.0	5.35	N	N	Inside-AD
PCP	0.57	0.0	10.58	N	N	Inside-AD
Poly(vinylbromide)	0.5	0.0	1.69	N	N	Inside-AD
PVC	0.54	0.0	2.6	N	N	Inside-AD
PVF	0.64	0.0	1.92	P	N	Inside-AD
PGA	0.73	0.0	6.53	N	N	Inside-AD
P4HB	0.59	0.0	5.42	N	N	Inside-AD
PLA	0.42	0.0	4.22	P	P	Inside-AD
Poly(3-butoxypropyleneoxide)	0.45	0.0	21.33	N	P	Inside-AD
Poly(epichlorohydrin)	0.39	0.0	5.87	N	P	Inside-AD
Poly(hexamethyleneglycol)	0.53	0.0	16.92	P	N	Inside-AD
PPG	0.39	0.0	3.87	P	P	Inside-AD
Poly(tetrahydrofuran)	0.57	0.0	2.78	P	N	Inside-AD
Poly(trimethyleneglycol)	0.29	0.0	5.05	P	P	Inside-AD
Polychloroprene	0.4	0.0	3.96	N	P	Inside-AD
Poly(1-bromo-1-butenylene)	0.0	0.0	0.0	P	P	Outside-AD
Poly(3-chlorostyrene)	0.4	0.0	6.04	N	P	Inside-AD
Poly(4-chlorostyrene)	0.44	0.0	6.21	P	P	Inside-AD
Poly(2-hydroxypropylmethacrylate)	0.47	0.0	15.77	N	N	Inside-AD
Poly(dimethylitaconate)	0.41	0.0	9.79	P	P	Inside-AD
Poly di(nbutyl)itaconate	0.45	10.35	40.83	P	P	Inside-AD
Poly(2,6-dimethyl-p-phenylene)oxide	0.46	0.0	13.9	P	N	Inside-AD
Poly(vinylacetate)	0.41	0.0	5.22	N	P	Inside-AD
Poly(vinylbenzoate)	0.43	0.0	10.96	P	P	Inside-AD
Poly(vinylformate)	0.5	0.0	2.64	N	N	Inside-AD
Poly(vinylsterate)	0.63	10.87	105.91	P	N	Inside-AD

Poly(vinylmethylketone)	0.45	0.0	7.92	N	P	Inside-AD
Poly(vinylphenylketone)	0.4	0.0	10.05	P	P	Inside-AD
Poly(vinylpyrrolidone)	0.54	0.0	7.58	N	N	Inside-AD
Poly(butylvinylthioether)	0.52	0.0	17.85	P	N	Inside-AD
Poly(methylvinylthioether)	0.47	0.0	3.45	P	N	Inside-AD
